# Role of lactylation modification in regulating lytic cell death

**DOI:** 10.3389/fonc.2026.1718636

**Published:** 2026-01-27

**Authors:** Xiaokang Zhang, Jing Luo, Zhengrong Zhang, Yang Yu, Jincan He, Zan Zuo, Ying An, Lingting Xun, Hua Jin, Jialong Qi, Jun Peng

**Affiliations:** 1 The First People’s Hospital of Yunnan Province, The Affiliated Hospital of Kunming University of Science and Technology, Kunming, China; 2 Pu’er People’s Hospital, The Affiliated Hospital of Kunming University of Science and Technology, Puer, China; 3 School of Medicine, Kunming University of Science and Technology, Kunming, Yunnan, China; 4 Yunnan Digestive Endoscopy Clinical Medical Center, Department of Gastroenterology, The First People’s Hospital of Yunnan Province, Kunming, Yunnan, China

**Keywords:** epigenetic regulation, lactylation, lytic cell death, post translational modification, therapeutic targeting

## Abstract

Historically, lactate has been regarded primarily as the terminal product of glycolysis. However, recent research has elucidated its critical function as an epigenetic regulator via lactylation modification. Lactylation represents a novel post-translational modification (PTM) characterized by the covalent attachment of lactyl groups to lysine residues on both histone and non-histone proteins. This modification influences protein function at transcriptional and post-translational stages, thereby forging a direct connection between lactate metabolism and epigenetic regulation. Lytic cell death (LCD) encompasses a group of inflammatory programmed cell death modalities characterized by the rupture of the plasma membrane, playing a pivotal role in the pathogenesis of various diseases. This review provides a comprehensive synthesis of recent advancements in understanding the regulatory axis between L-lactate–induced lysine lactylation (KL-la) and LCD in disease contexts. Emerging evidence suggests that KL-la modulates several LCD subtypes, including pyroptosis, ferroptosis, and NETosis, thereby influencing disease progression and clinical outcomes. Notably, the regulatory effects of KL-la are highly context-dependent. Within the tumor microenvironment, KL-la primarily inhibits LCD, thereby promoting tumor cell survival. Conversely, in non-tumor conditions such as inflammation and ischemic injury, KL-la frequently enhances LCD, leading to increased tissue damage. This review also underscores therapeutic strategies that target lactate metabolism and KL-la-related enzymes to modulate LCD. Future interventions must incorporate the pathological context, cell-type specificity, and molecular targets. The advancement of context-responsive precision strategies, such as microenvironment-activated prodrugs or cell-specific delivery systems, will be crucial for the realization of safe and effective targeted therapies.

## 1 Introduction

Lactate, traditionally considered a metabolic byproduct of glycolysis under hypoxic conditions, is increasingly recognized as a crucial energy carrier and a signaling molecule involved in intracellular signal transduction (1). Moreover, lactate influences regulatory processes at both transcriptional and protein functional levels (2–4). In 2019, Zhao et al. identified the histone-modifying activity of lactate, thereby introducing lactylation as a novel epigenetic regulatory mechanism (5). Lactylation can be broadly categorized into histone and non-histone forms based on substrate specificity (6). From a structural standpoint, lysine lactylation comprises three distinct chemical variants: L-lactylation (KL-la), D-lactylation (KD-la), and lysine 2-hydroxypropionylation (Kce) (7, 8). Mechanistically, lactylation can occur via enzymatic processes, as exemplified by KL-la, or through non-enzymatic reactions, such as KD-la and Kce (9). Among these modifications, KL-la adheres to a canonical regulatory paradigm involving writers, readers, and erasers, which is mediated by lactyltransferases, reader proteins, and delactylases. This intricate regulatory network facilitates KL-la’s role as a critical mechanistic link between lactate metabolism and epigenetic regulation within disease contexts (10, 11).

Cell death is a fundamental biological process essential for regulating organismal development, maintaining homeostasis, and participating in pathological responses (12). Dysregulation of cell death is intricately linked to the initiation and progression of various diseases (13). Based on underlying mechanisms, cell death can be broadly categorized into accidental cell death (ACD) and regulated cell death (RCD). RCD can be further subdivided, according to morphological characteristics and inflammatory outcomes, into non-lytic cell death (NLCD) and lytic cell death (LCD) (14). LCD is distinguished by the rupture of the plasma membrane and the induction of robust inflammatory responses (15, 16). The major forms of LCD include pyroptosis (17), necroptosis (18), ferroptosis (14), and neutrophil extracellular trap-mediated death (NETosis) (19).

Accumulating evidence suggests that KL-la plays a pivotal role in the regulation of LCD. KL-la can precisely modulate LCD activation by either directly influencing core proteins involved in pyroptosis, ferroptosis, and NETosis or by regulating their transcriptional programs and post-translational modification states (20). Numerous studies have demonstrated that KL-la affects disease progression and prognosis through its regulation of LCD. Notably, its regulatory effects exhibit contrasting patterns in tumor versus non-tumor contexts. In tumors, KL-la often suppresses LCD in cancer cells, thereby facilitating tumor growth and survival (21–23). Conversely, in non-tumor diseases, KL-la tends to enhance LCD in normal cells, which exacerbates tissue damage and accelerates disease progression (24, 25). Although targeting lactate production or modifying KL-la to modulate LCD has been proposed as a potential therapeutic strategy (26, 27), its efficacy is highly context-dependent. Consequently, the development of preventive and therapeutic strategies must carefully consider the specific disease context.

In conclusion, KL-la exhibits bidirectional regulatory effects on LCD, influencing disease progression in both tumor and non-tumor contexts. Despite this, a comprehensive and systematic synthesis of the KL-la–LCD–disease regulatory axis and its underlying mechanisms remains absent. Consequently, this review endeavors to systematically summarize the regulatory roles of both histone and non-histone KL-la in LCD across various disease contexts, providing an in-depth analysis of the molecular mechanisms through which KL-la modulates disease progression by influencing different LCD subtypes. Furthermore, we conduct a comprehensive review of therapeutic strategies targeting lactate metabolism and lactylation-related enzymes to regulate LCD, encompassing traditional Chinese medicine, chemical drugs, and small-molecule inhibitors. Collectively, this review aspires to offer novel perspectives and a theoretical framework for the development of future clinical interventions.

## 2 Lactate metabolism and lactylation modification

### 2.1 Historical perspectives on lactate and lactylation

The discovery of lactate dates back to the eighteenth century. In 1780, Carl Wilhelm Scheele was the first to isolate lactate from sour milk. Later, in 1843, Johann Joseph Scherer identified a correlation between lactate concentrations in human blood and pathological conditions, providing early evidence of lactate’s involvement in pathophysiological processes (28). In 1873, Johannes Wislicenus further clarified the chemical structure of lactate, thus laying a molecular foundation for future biochemical research (2). Throughout the twentieth century, the understanding of lactate’s biological functions has progressively expanded. In 1972, Otto Warburg introduced the Warburg effect, demonstrating that tumor cells preferentially utilize glycolysis even in the presence of oxygen, thereby underscoring lactate’s central role in tumor metabolism (29). Technological advancements, such as the introduction of FDG PET in 1980, facilitated the *in vivo* visualization of lactate-related metabolic activity (30). In 1985, the lactate shuttle theory was proposed, which significantly altered the perception of lactate from merely a metabolic waste product to an essential signaling molecule (31).

**Figure 1:**
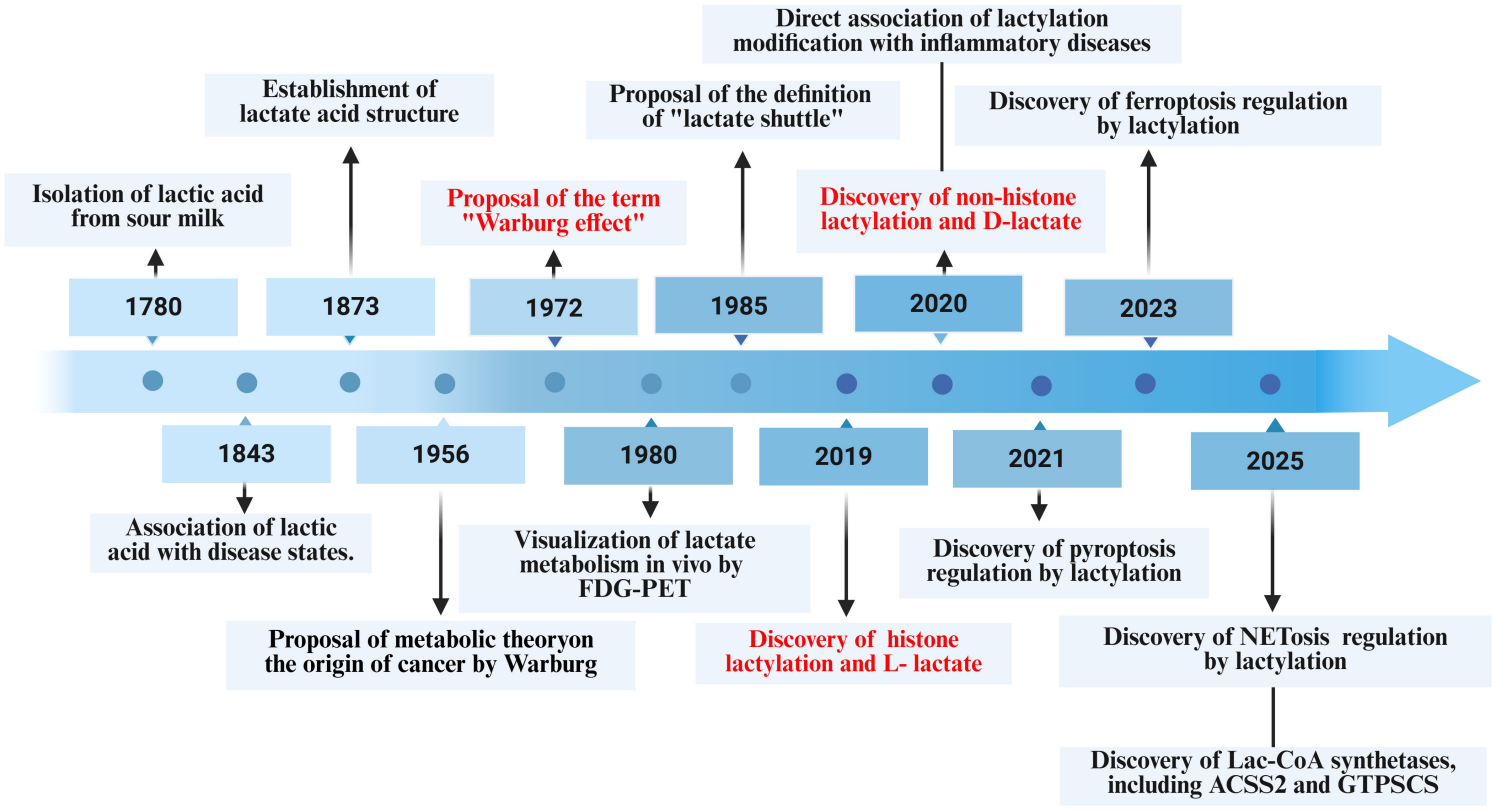
Timeline of lactate metabolism and lactylation modification research. Since the initial discovery of lactate in 1780, the relationship between lactate and disease pathogenesis has been progressively elucidated. The establishment of the “Warburg effect” and lactate shuttle theory significantly advanced our understanding of the biological functions of lactate. The groundbreaking discovery of lactylation in 2019 bridged lactate metabolism with epigenetic regulation. Recent advances have extensively characterized the roles of lactate and lactylation in disease pathogenesis and their regulatory effects on lytic cell death. FDG-PET, 18F-fluorodeoxyglucose-positron emission tomography; Lac-CoA, lactyl-coenzyme A; ACSS2, acyl-CoA synthetase short-chain family member 2; GTPSCS, GTP-specific succinyl-CoA synthetase.

In recent years, accumulating studies have demonstrated that lactylation plays an important regulatory role in LCD. In 2021, Zhao et al. first reported that lactylation regulates pyroptosis, providing initial evidence for the involvement of this modification in LCD pathways (32). In 2023, Yu et al. further elucidated the molecular mechanisms through which lactylation modulates ferroptosis (33). In 2025, Zhu et al. constructed a complete signaling axis by which lactylation regulates neutrophil NETosis, thereby establishing a comprehensive framework linking lactylation to this distinct form of LCD (34) (**Figure 1**).

### 2.2 Classification of lactylation

Lactylation represents a post-translational modification (PTM) characterized by the covalent attachment of acyl groups to lysine residues, occurring via both enzymatic and non-enzymatic mechanisms. This modification can be categorized into histone lactylation and non-histone lactylation, depending on the substrates involved. Histone lactylation primarily influences gene transcription by modulating the interactions between histones and DNA, whereas non-histone lactylation predominantly affects the activity and function of target proteins through direct modification. Collectively, these two forms of lactylation are implicated in a diverse array of biological processes (35, 36). Chemically, lactylation encompasses three distinct isomeric forms: lysine L-lactylation (KL-la), lysine D-lactylation (KD-la), and lysine 2-hydroxypropionylation (Kce) (37).

**Figure 2:**
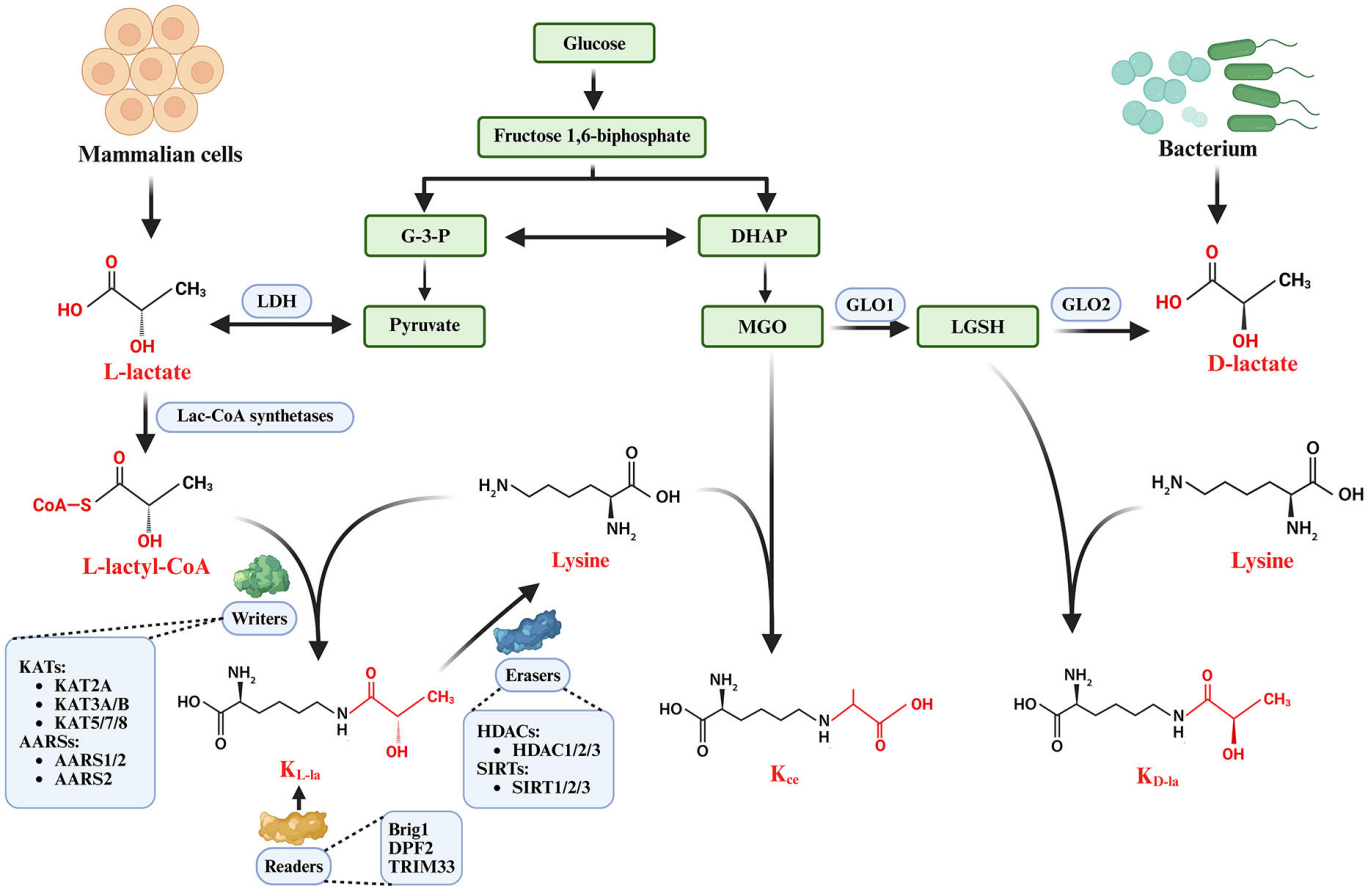
Classification and regulatory mechanisms of lactylation modifications. Lactate comprises two primary isomers: L-lactate and D-lactate. In eukaryotic cells, glucose undergoes glycolytic conversion to pyruvate, which is subsequently catalyzed by LDH to generate L-lactate. In prokaryotic organisms, bacteria utilize GLO1 and GLO2 to convert MGO into D-lactate. Based on substrate specificity and formation mechanisms, lactylation modifications are categorized as K_L-la_, K_ce_, and K_D-la_. L-lactylation occurs through enzymatic processes and follows the classic “writer-eraser-reader” regulatory paradigm. K_ce_ results from direct MGO-lysine interactions, whereas K_D-la_ is mediated through nonenzymatic D-lactate reactions. G-3-P, glyceraldehyde-3-phosphate; DHAP, dihydroxyacetone phosphate; LDH, lactate dehydrogenase; MGO, methylglyoxal; GO1, glyoxalase 1; GO2, glyoxalase 2; LGSH, lactoylglutathione; KL-la, L-lactylation; KD-la, D-lactylation; Kce, carboxyethylation; KAT2A, lysine-acetyltransferase 2A; KAT3A, lysine-acetyltransferase 3A; KAT3B, lysine-acetyltransferase 3B; KAT5, lysine-acetyltransferase 5; KAT7, lysine-acetyltransferase 7; KAT8, lysine-acetyltransferase 8; HDAC1, histone deacetylase 1; HDAC2, histone deacetylase 2; HDAC3, histone deacetylase 3; SIRT1, sirtuin 1; SIRT2, sirtuin 2; SIRT3, sirtuin 3; Brig1, Brahma-related gene 1; DPF2, D4, zinc and double PHD fingers family 2; TRIM33, tripartite motif-containing 33.

The mechanisms underlying their formation exhibit significant differences. KL-la is predominantly produced through enzymatic reactions facilitated by L-lactate, which is derived from glycolysis in eukaryotic cells, including those of mammals. Conversely, KD-la primarily originates from D-lactate, which is generated by prokaryotic organisms such as bacteria via non-enzymatic pathways (37). Kce is synthesized through the direct reaction of methylglyoxal with lysine residues (38). Therefore, from a mechanistic perspective, lactylation can be classified into enzymatic lactylation, as exemplified by KL-la, and non-enzymatic lactylation, as represented by KD-la and Kce (**Figure 2**).

### 2.3 Enzymatic regulators and regulatory mechanisms of KL-la

KL-la follows a canonical dynamic regulatory paradigm involving writers, readers, and erasers (**Supplementary Table 1**). This modification uses lactyl-CoA as its direct substrate, which can be synthesized by enzymes such as acyl-CoA synthetase short-chain family member 2(ACSS2) and GTP-specific succinyl-CoA synthetase(GTPSCS) (39, 40).

Enzymes known as “writers” facilitate the transfer of lactyl groups to lysine residues. Currently, identified writer enzymes are primarily categorized into two groups. The first group comprises members of the lysine acetyltransferase family, such as p300 (41, 42), CBP (43), GCN5 (44), MOF (45, 46), TIP60 (47), and HBO1 (48). The second group includes alanyl tRNA synthetases, specifically AARS1 and AARS2, which have the capability to directly utilize lactate as a substrate for catalyzing lactylation (49–52). Emerging evidence also indicates that HDAC6 may exhibit lactyltransferase activity (53). Enzymes referred to as “erasers” are responsible for the removal of lactyl groups from modified proteins, primarily encompassing histone deacetylases HDAC1-3 (37, 47, 53) and sirtuin family deacetylases SIRT1-3 (54, 55). “Reader” proteins are tasked with the specific recognition and binding of lactylated lysine residues. Currently, only a limited number of lactylation readers have been identified, including Brg1 (56), DPF2 (59, and TRIM33 (57) (**Figure 2**, **Supplementary Table 1**).

### 2.4 Site selectivity of distinct KL-la enzymes toward histone lysine residues

Histone KL-la demonstrates significant site selectivity for specific residues, such as H3K9, H3K18, and H4K12. This selectivity is orchestrated by the coordinated actions of the writer, reader, and eraser enzymatic network, serving as a crucial determinant of the functional diversity of histone KL-la. At the writer level, lactyltransferases exhibit distinct site preferences. For instance, HBO1, also referred to as KAT7, preferentially catalyzes H3K9la (48). KAT2A and KAT2B predominantly facilitate the formation of H3K18la (58), whereas KAT5 and KAT8 are more closely associated with the establishment of H4K12la (57). At the reader level, recognition proteins also display site selectivity. DPF2 specifically recognizes H3K14la (59, 60), while TRIM33 and Brg1 preferentially bind to H3K18la (61). At the eraser level, delactylating enzymes similarly exhibit residue preference. HDAC1 and HDAC3 significantly reduce the levels of H4K5la and H4K12la (62), whereas SIRT2 more effectively removes lactylation at H3K18 and related sites (63).

Collectively, site selection of histone KL-la is not stochastic but is governed by the coordinated regulation of writer enzymes, eraser enzymes, and reader proteins. The precise molecular mechanisms underlying this selectivity remain to be systematically elucidated.

## 3 Regulatory roles and mechanisms of KL-la in LCD

LCD encompasses a category of regulated cell death (RCD) modalities distinguished by the loss of plasma membrane integrity, the release of intracellular contents, and the significant liberation of damage-associated molecular patterns (DAMPs), which typically provoke pronounced inflammatory responses. According to the Nomenclature Committee on Cell Death (NCCD), pyroptosis and necroptosis are considered archetypal forms of LCD, with the rupture of the plasma membrane serving as the defining terminal event (13). Emerging evidence indicates that both ferroptosis and NETosis result in membrane disruption, accompanied by the release of extensive inflammatory mediators (64, 65). Although these processes are not traditionally classified under the category of lytic cell death (LCD), they are included in this analysis to enable a thorough exploration of KL-la function across various membrane-disruptive cell death pathways, with subsequent mechanistic distinctions to be elucidated. This chapter provides a systematic examination of the regulatory roles and molecular mechanisms by which KL-la modulates the aforementioned LCD subtypes, including pyroptosis, ferroptosis, and NETosis.

### 3.1 Regulatory role and mechanism of KL-la on pyroptosis

Pyroptosis is a prototypical form of lytic cell death (LCD) characterized by cellular swelling, the formation of pores in the plasma membrane, cytolysis, and the release of pro-inflammatory factors (66). This process is predominantly mediated by the Gasdermin protein family, with Gasdermin D (GSDMD) acting as the crucial effector in inflammasome-dependent pyroptosis (67–69). Pyroptosis can be categorized into canonical and non-canonical pathways based on the mechanisms of activation. The canonical pathway involves the activation of Caspase-1 via the inflammasome, leading to the cleavage of GSDMD and the maturation of interleukin-1β (IL-1β) and interleukin-18 (IL-18) (70). In contrast, the non-canonical pathway is initiated by the direct activation of Caspase-4, -5, or -11 by intracellular lipopolysaccharide, which induces GSDMD cleavage and membrane pore formation, with potential amplification of the inflammatory response through subsequent inflammasome activation (71).

In this context, the inflammasome operates as an initiating switch within the canonical pathway and serves as an inflammatory amplifier in the non-canonical pathway, collectively orchestrating the execution of pyroptosis. Recent evidence indicates that lactylation modification regulates pyroptosis through two mechanisms: epigenetic regulation and protein functional modulation. At the histone KL-la level, these modifications alter the transcriptional programs of genes associated with inflammation. For example, H3K18la enhances the expression of NLRP3 and high mobility group box 1 (HMGB1), thereby facilitating inflammasome activation (72, 73). Conversely, H4K12la promotes pyroptosis by increasing NEK7 expression (74). At the non-histone level, lactylation directly modifies and regulates essential proteins within the pyroptotic pathway. Specifically, NLRP3-K166la enhances its interaction with ASC, promoting inflammasome assembly (75). Additionally, NEDD4-K33la inhibits ubiquitination-mediated degradation of Caspase-11, thereby activating non-canonical pyroptosis (76). Furthermore, lactylation of SOX10 upregulates the expression of GSDMD and Caspase-1, thereby advancing pyroptotic progression (77).

Although it has been confirmed that KL-la modifications, including H3K18la and H4K12la, play a role in the regulation of pyroptosis, their dynamic mechanisms and functional specificity require further investigation. Firstly, the temporal dynamics and functional differentiation of specific lactylation sites during key pyroptotic stages, such as inflammasome priming and GSDMD cleavage, are not yet fully understood. Secondly, it remains uncertain whether identical sites exhibit varying functionalities across different pyroptotic pathways. Thirdly, the potential cooperation between lactylation and other post-translational modifications (PTMs), such as acetylation and ubiquitination, through competitive site occupancy in regulating critical events like GSDMD activation, needs to be validated. Future research utilizing site-specific mutagenesis, modification omics, and time-resolved proteomics is essential to elucidate the dynamic regulatory landscape of lactylation in pyroptosis and to explore its interaction networks with other PTMs.

### 3.2 Regulatory role and mechanisms of KL-la in ferroptosis

Ferroptosis constitutes an iron-dependent mode of regulated cell death (RCD) initiated by the accumulation of lipid peroxides, and is marked by the reduction of mitochondrial cristae and disruption of the outer mitochondrial membrane (78). The fundamental mechanistic characteristics include dysregulation of iron metabolism, inactivation of glutathione peroxidase 4 (GPX4), and lipid peroxidation mediated by acyl-CoA synthetase long-chain family member 4 (ACSL4) (79, 80).

Accumulating evidence demonstrates that KL-la exerts precise, context-dependent regulatory control over critical ferroptotic events through histone and non-histone modification modalities. At the level of iron metabolism, histone lysine lactylation, exemplified by H3K18la, upregulates the expression of ferritin heavy chain 1 (FTH1), thereby enhancing iron sequestration and subsequently inhibiting ferroptosis (23).At the level of antioxidant defense, KL-la-mediated regulation of GPX4 demonstrates notable bidirectionality. In non-tumorous diseases, H3K18la suppresses GPX4 transcription to promote ferroptosis (25), whereas in malignant conditions, it enhances GPX4 expression to provide resistance against ferroptosis (33). At the lipid peroxidation stage, H3K18la facilitates the upregulation of ACSL4 transcription, thereby increasing the generation of lipid peroxidation substrates and subsequently promoting ferroptosis (81). Concurrently, at the post-translational level, the non-histone protein KL-la augments the stability of the ACSL4 protein, collectively advancing the execution of ferroptosis (24).

In summary, KL-la regulates cellular ferroptosis through context-dependent bidirectional modulation of essential ferroptotic proteins, including FTH1, GPX4, and ACSL4, at both transcriptional and post-translational levels. However, significant mechanistic questions remain unanswered. It is still unclear whether histone modifications at sites beyond H3K18la, such as H3K9 and H3K27, play a role in the regulation of ferroptosis. Additionally, the co-regulatory factors that influence the bidirectional regulation of GPX4 have yet to be identified. Furthermore, the potential direct modification of other critical proteins, such as SLC7A11 and FSP1, by KL-la requires further investigation. Lastly, the temporal dynamics of KL-la modifications throughout the progression of ferroptosis have not been systematically characterized.

### 3.3 Regulatory role and mechanisms of KL-la in NETosis

NETosis is a distinct form of regulated cell death (RCD) characterized by the rupture of the plasma membrane and the subsequent release of neutrophil extracellular traps (NETs) (82). NETs are composed of web-like structures formed by decondensed chromatin intertwined with granular proteins such as myeloperoxidase (MPO) and neutrophil elastase (NE) (83). The execution of NETosis relies on two primary pathways: the citrullination of histones mediated by peptidylarginine deiminase 4 (PAD4) and the intranuclear proteolysis of histones mediated by NE. PAD4 initiates chromatin decondensation by reducing the affinity between histones and DNA, while NE further hydrolyzes histones. These processes work synergistically to facilitate the release of NETs (84, 85).

In inflammatory or tumor microenvironments, enhanced glycolytic activity in neutrophils results in the accumulation of lactate, which subsequently induces modifications in histone lactylation. Specifically, lactate prompts an increase in H3K18la levels, which weakens the electrostatic interactions between histones and DNA. This alteration facilitates the transition of chromatin from a compact to a relaxed state, thereby providing the necessary structural framework for the release of neutrophil extracellular traps (NETs) (86). Importantly, chromatin relaxation mediated by H3K18la significantly enhances PAD4-dependent histone citrullination and NE-mediated histone proteolysis. This synergistic interaction facilitates chromatin decondensation, thereby promoting the execution of NETosis (86).

Although preliminary insights have been gained into the promotion of NETosis by KL-la, significant mechanistic gaps remain. It is still unclear whether there are additional specific modification sites beyond H3K18la. Furthermore, it is yet to be determined whether KL-la directly influences the core enzymatic activities of PAD4 and NE. Future research should focus on systematically mapping the lactylation landscapes associated with NETosis and dissecting the functional and regulatory networks that govern key modification sites and specific enzymes. This approach is essential for a comprehensive understanding of this metabolic-immune regulatory axis.

## 4 Roles of histone KL-la in disease associated regulation of LCD

Histone KL-la represents a pivotal modification bridging cellular metabolism and epigenetics, directly governing gene transcription through covalent modification of histone lysine residues (11). Accumulating evidence demonstrates that KL-la orchestrates central roles in tumor and non-tumor disease progression by precisely modulating LCD processes including pyroptosis, ferroptosis, and NETosis (5, 87). The KL-la–LCD–disease regulatory axis demonstrates significant context-dependence, wherein identical modifications, such as H3K18la and H4K12la, exert contrasting effects across different pathological contexts. In non-tumorous diseases, these modifications generally enhance LCD and worsen tissue damage, whereas in tumor settings, they often inhibit LCD to support tumor survival and progression. This bidirectional regulatory mechanism is influenced by a complex interplay of factors, including cell type, microenvironmental signals, and the landscape of co-existing modifications. This chapter systematically elucidates the molecular mechanisms by which histone KL-la regulates LCD across various disease contexts. Through a comparative analysis of the mechanistic parallels and divergences, it offers an integrative framework for developing therapeutic strategies targeting this axis (**Table 1**).

**Table 1:** Histone K_L-la_-mediated regulation of LCD in diseases.

Lactylation site	Target gene	Effect on LCD		Disease	Effect on disease	Reference
H3K18	HMGB1	Pyroptosis	↑	CIRI	Exacerbation	(74)
	NOD2			BRE		(88)
	YTHDF3		↓	SEV-CI	Inhibition	(89)
	ALKBH5			GSD-IBD	Exacerbation	(90)
	NLRP3			UC	Inhibition	(32)
H4K12	NEK7	Pyroptosis	↑	AD	Exacerbation	(75)
H3K18	ATF3/ATF4/CHAC1	Ferroptosis	↑	NASH	Exacerbation	(25)
	ACSL4			IVDD		(24)
	METTL3			SILI		(81)
	AIM2		↓	LC		(21)
	NSF1			HCC		(22)
	ZFP64			TNBC		(23)
	HIF1α			PCa		(33)
H4K12	GCLC	Ferroptosis	↓	CRC	Exacerbation	(91)
H3K14	TFRC	Ferroptosis	↑	SI-ARDS	Exacerbation	(90)
H3K18	HMGB1	NETosis	↑	SAKI	Exacerbation	(92)

H3K18, histone H3 lysine 18; H4K12, histone H4 lysine 12; H3K14, histone H3 lysine 14; HMGB1, high-mobility group box 1; NOD2, nucleotide-binding oligomerization domain-containing 2; YTHDF3, YTH N6-methyladenosine RNA-binding protein 3; ALKBH5, AlkB homolog 5 RNA demethylase; NLRP3, NOD-like receptor family pyrin domain-containing 3; NEK7, NIMA-related kinase 7; ATF3, activating transcription factor 3; ATF4, activating transcription factor 4; CHAC1, ChaC glutathione-specific gamma-glutamylcyclotransferase 1; ACSL4, acyl-CoA synthetase long-chain family member 4; METTL3, methyltransferase-like 3; AIM2, absent in melanoma 2; NSF1, N-ethylmaleimide-sensitive factor 1; ZFP64, zinc finger protein 64; HIF1α, hypoxia-inducible factor 1 alpha; GCLC, glutamate-cysteine ligase catalytic subunit; TFRC, transferrin receptor; CIRI, cerebral ischemia–reperfusion injury; BRE, bilirubin encephalopathy; SEV-CI, sevoflurane-induced cognitive impairment; GSD-IBD, glycogen storage disease–associated inflammatory bowel disease; UC, ulcerative colitis; AD, Alzheimer’s disease; NASH, nonalcoholic steatohepatitis; IVDD, intervertebral disc degeneration; SILI, sepsis-induced lung injury; LC, lung cancer; HCC, hepatocellular carcinoma; TNBC, triple-negative breast cancer; PCa, prostate cancer; CRC, colorectal cancer; SI-ARDS, sepsis-induced acute respiratory distress syndrome; SAKI, sepsis-associated acute kidney injury.

### 4.1 Histone KL-la regulation of pyroptosis in non-tumor diseases

**Figure 3:**
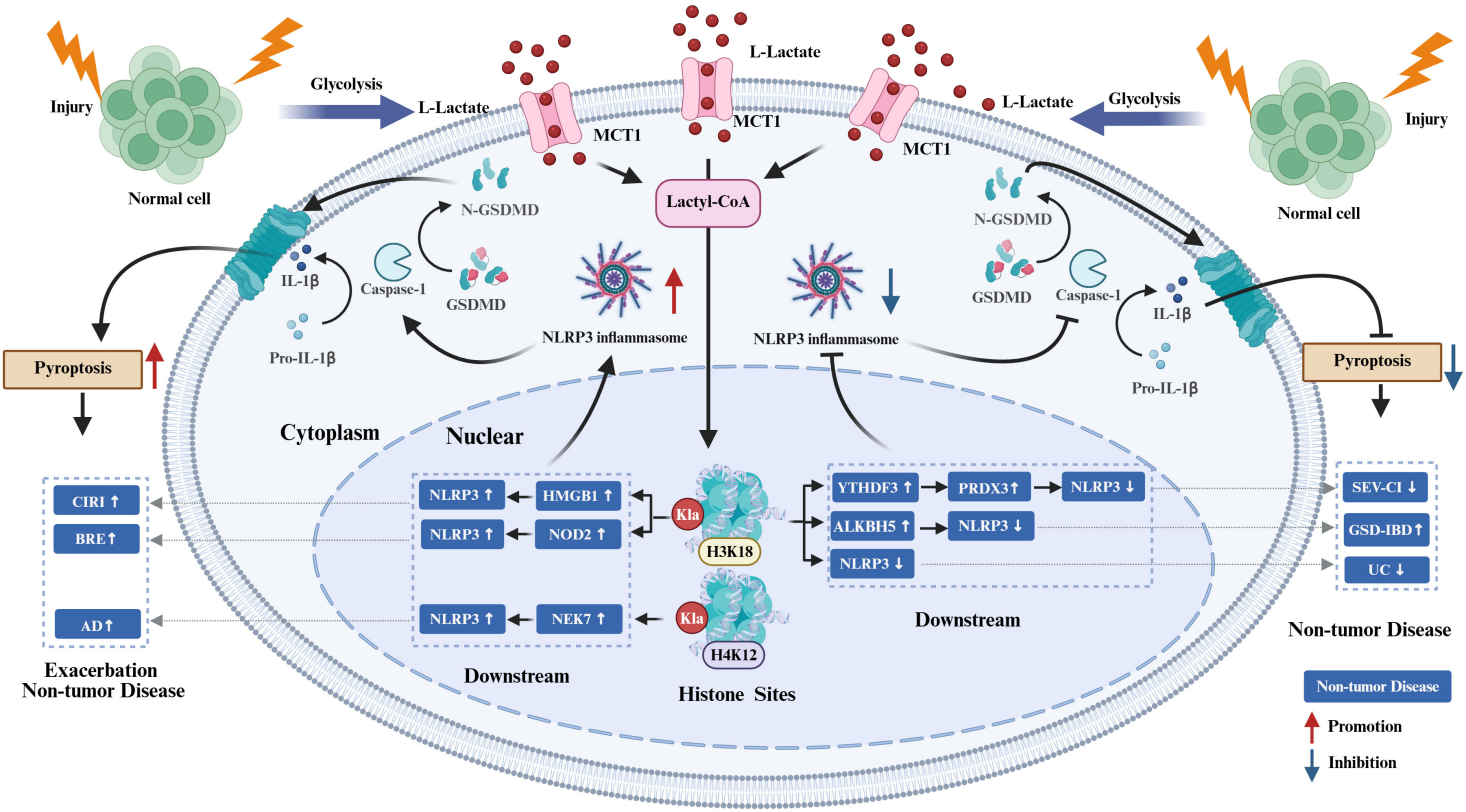
Histone KL-la regulation of pyroptosis in non-tumor diseases. Under injury, stress, and inflammatory conditions, glycolytic enhancement drives L-lactate accumulation that undergoes MCT1-mediated transport and lactyl-CoA conversion to induce H3K18 and H4K12 lactylation. H3K18la/H4K12la modulate downstream effectors including HMGB1, NOD2, NEK7, YTHDF3, ALKBH5, PRDX3, and NLRP3 to influence NLRP3 inflammasome activation, caspase-1 activation, and GSDMD cleavage, thereby bidirectionally regulating pyroptosis. Within CIRI, BRE, and AD, histone KL-la promotes pyroptosis and intensifies disease progression. In GSD-IBD, H3K18la suppresses macrophage pyroptosis yet exacerbates pathological outcomes. Conversely, in SEV-CI and UC, H3K18la confers protection through pyroptosis inhibition. Red arrows denote promotional effects while blue arrows indicate inhibitory actions. H3K18, histone H3 lysine 18; H4K12, histone H4 lysine 12; H3K18la, histone H3 lysine 18 lactylation; H4K12la, histone H4 lysine 12 lactylation; KL-la, lysine lactylation; MCT1, monocarboxylate transporter 1; lactyl-CoA, lactyl-coenzyme A; HMGB1, high mobility group box 1; NOD2, nucleotide-binding oligomerization domain-containing protein 2; NEK7, NIMA-related kinase 7; YTHDF3, YTH N6-methyladenosine RNA binding protein F3; ALKBH5, alkB homolog 5; PRDX3, peroxiredoxin 3; NLRP3, NOD-like receptor family pyrin domain containing 3; caspase-1, cysteine-aspartic protease 1; GSDMD, gasdermin D; CIRI, cerebral ischemia-reperfusion injury; BRE, bilirubin encephalopathy; AD, Alzheimer’s disease; GSD-IBD, glycogen storage disease-associated inflammatory bowel disease; SEV-CI, sevoflurane-induced cognitive impairment; UC, ulcerative colitis.

In various non-tumor pathologies, the regulation of pyroptosis by histone KL-la demonstrates significant context-dependence. Recent evidence suggests that histone KL-la modulates both the intensity and directionality of cellular pyroptosis in neurological disorders and inflammatory bowel disease (IBD) by specifically targeting different components of the inflammasome-caspase-Gasdermin axis. This modulation subsequently alters local inflammatory responses and impacts disease outcomes, as illustrated in **Figure 3**.

#### 4.1.1 Histone KL-la regulation of pyroptosis in neurological diseases

In the context of neurological diseases, inflammation-induced enhancement of glycolysis and subsequent lactate accumulation lead to histone KL-la modifications, which transcriptionally reprogram signaling pathways associated with pyroptosis, significantly influencing the progression of neuroinflammation and the trajectory of the disease. Contemporary research primarily concentrates on the regulation of the canonical NLRP3-caspase-1-GSDMD pathway by KL-la, uncovering distinct site-specificity that varies according to cell type and disease model.

In models of cerebral ischemia-reperfusion injury (CIRI), bilirubin encephalopathy (BRE), and Alzheimer’s disease (AD), KL-la exhibits pro-inflammatory and pro-pyroptotic characteristics. Specifically, in CIRI, neuronal lactate-mediated H3K18la directly activates the NLRP3/caspase-1/GSDMD signaling pathway by upregulating HMGB1 transcription. This activation promotes neuronal pyroptosis and exacerbates cerebral tissue damage (73). In a similar manner, increased levels of hippocampal H3K18la in the BRE context facilitate caspase-1-mediated astrocytic pyroptosis through the upregulation of nucleotide-binding oligomerization domain-containing protein 2 (NOD2) and the activation of the MAPK/NF-κB signaling pathway (93). Additionally, in Alzheimer’s disease (AD) models, microglial H4K12la enhances inflammasome activation by elevating the expression of NIMA-related kinase 7 (NEK7), a pivotal factor in the assembly of the NLRP3 inflammasome, thereby triggering neuroinflammatory responses and contributing to disease progression (74).These findings demonstrate that specific histone lactylation sites including H3K18la and H4K12la synergistically amplify NLRP3 inflammasome activity across distinct central nervous system cell types through regulation of differential downstream targets, intensifying pyroptotic signaling and precipitating adverse disease outcomes. Nevertheless, histone KL-la regulation of pyroptosis is not unidirectional. In sevoflurane-induced cognitive impairment (SEV-CI) models, repetitive sevoflurane exposure reduces hippocampal lactate levels and H3K18la, subsequently attenuating the YTHDF3-PRDX3 antioxidant axis and culminating in excessive NLRP3 inflammasome activation alongside microglial pyroptosis (88). This counterexample reveals that H3K18la can suppress pyroptosis and preserve neural function by sustaining antioxidant homeostasis under specific pathological contexts, underscoring its functional plasticity and pathological context-dependence.

The regulatory influence of histone KL-la, specifically H3K18la, on pyroptosis appears to be collectively determined by the inflammatory states that initiate the disease and the chromatin landscapes specific to the disease model. Significant knowledge gaps remain in the field. In addition to H3K18la and H4K12la, the roles of other histone KL-la sites in neurological diseases have yet to be investigated. Furthermore, the cell type-specific functions of identical KL-la sites across neurons, microglia, and astrocytes require further elucidation. Current research predominantly focuses on canonical pyroptotic pathways, thereby neglecting the regulation of non-canonical pyroptosis by histone KL-la modifications. Future research endeavors should utilize epigenetic editing platforms, such as CRISPR-dCas9, for the site-specific manipulation of KL-la to elucidate the distinct functions of various sites. Concurrently, conditional gene ablation strategies should be employed to investigate the functional implications of these modifications across different neural cell populations.

#### 4.1.2 Histone KL-la regulation of pyroptosis in inflammatory bowel disease

In the context of inflammatory bowel disease (IBD), the regulation of histone KL-la in macrophages plays a pivotal role as an epigenetic determinant of intestinal inflammatory pathways. Notably, identical effects that suppress pyroptosis can lead to contrasting disease outcomes, depending on the functional states of macrophages within different pathological contexts.

In glycogen storage disease-associated IBD (GSD-IBD), glucose-6-phosphate transporter (G6PT) deficiency drives enhanced macrophage glycolysis and lactate accumulation, subsequently inducing H3K18la. This modification suppresses pyroptosis by upregulating the N6-methyladenosine demethylase ALKBH5, which destabilizes NLRP3 mRNA (89). However, during intestinal homeostasis, macrophage pyroptosis is requisite for commensal microbes and pathobionts clearance. Consequently, sustained pyroptosis inhibition paradoxically attenuates immune surveillance, fostering dysbiosis and chronic inflammation. Conversely, in dextran sulfate sodium (DSS)-induced acute ulcerative colitis (UC) models, lactate accumulation similarly induces H3K18la-mediated NLRP3 suppression and macrophage pyroptosis inhibition, yet this constrains excessive inflammatory cascades to mitigate intestinal mucosal injury (32). This divergence arises from macrophages existing in a hyperactivated pro-inflammatory state during acute inflammatory peaks, during which pyroptosis extensively releases factors such as IL-1β, thereby further exacerbating tissue destruction. These contrasting scenarios reveal that H3K18la-mediated pyroptosis suppression generates opposite pathological outcomes in GSD-IBD macrophages operating under immune surveillance imperatives versus DSS-UC macrophages functioning amid inflammatory destruction. This paradigm vividly exemplifies the context-dependence governing histone KL-la regulation, wherein biological significance is dictated by the functional status of modified cells within specific microenvironmental niches.

Critical emerging questions involve identifying the upstream signals that dictate the functional divergence of macrophages and determining whether these signals operate through chromatin landscape remodeling or alterations in co-modification patterns. Furthermore, current investigations primarily focus on macrophages, leaving the roles of KL-la in pyroptosis of other intestinal cell populations, such as epithelial cells and T cells, largely unexplored. Future research must integrate cell type-specific epigenomic profiling with genetic editing techniques to elucidate the functional logic of KL-la within intact microenvironmental contexts, thereby laying the groundwork for context-selective therapeutic target.

### 4.2 Histone KL-la regulation of ferroptosis in diseases

**Figure 4:**
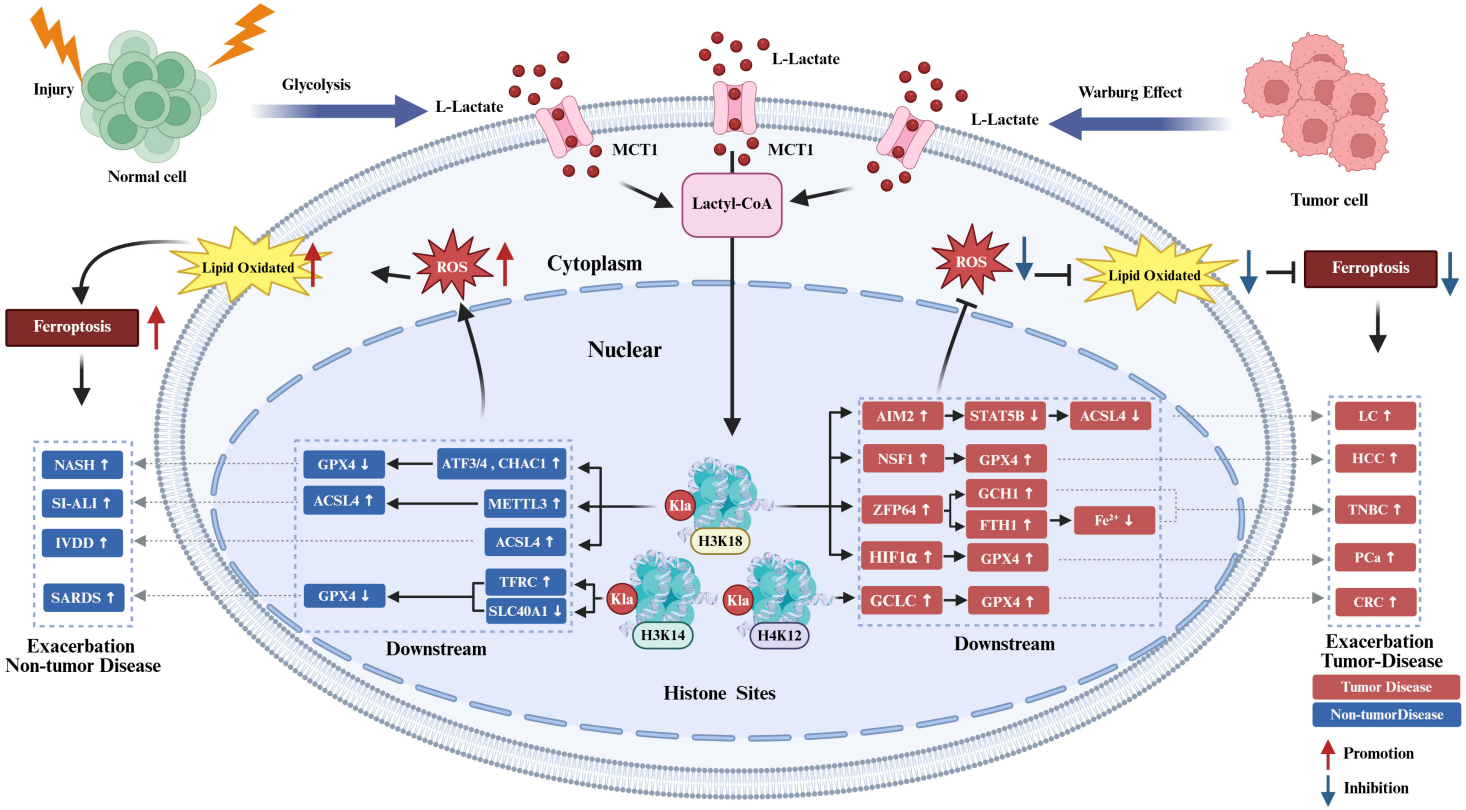
Histone KL-la regulation of ferroptosis in diseases. In non-tumor diseases, injury, stress, or inflammatory stimuli enhance glycolysis and elevate L-lactate levels, which undergoes MCT1-mediated transport and conversion to lactyl-CoA to induce histone lactylation at H3K18, H3K14, and H4K12 sites. These lactylation modifications promote lipid peroxidation and ROS accumulation through regulation of downstream molecules including ATF3/4-CHAC1, METTL3, ACSL4, and TFRC, thereby enhancing ferroptosis and aggravating progression of non-tumor diseases including NASH, SI-ALI, IVDD, and SARDS. Conversely, within tumor cells, the Warburg effect-driven persistent lactate-enriched environment similarly promotes histone lactylation, yet predominantly upregulates molecules including AIM2, NFS1, ZFP64, HIF-1α, and GCLC to enhance GPX4, GCH1, and FTH1 expression, reducing ROS, labile iron, and lipid peroxidation levels to suppress ferroptosis and promote progression of tumors including LC, HCC, TNBC, PCa, and CRC. Red and blue colors represent tumor and non-tumor disease contexts, respectively; upward arrows indicate promotion while downward arrows indicate inhibition. H3K18, histone H3 lysine 18; H3K14, histone H3 lysine 14; H4K12, histone H4 lysine 12; H3K18la, histone H3 lysine 18 lactylation; H3K14la, histone H3 lysine 14 lactylation; H4K12la, histone H4 lysine 12 lactylation; KL-la, lysine lactylation; MCT1, monocarboxylate transporter 1; lactyl-CoA, lactyl-coenzyme A; ROS, reactive oxygen species; ATF3, activating transcription factor 3; ATF4, activating transcription factor 4; CHAC1, glutathione-specific gamma-glutamylcyclotransferase 1; METTL3, methyltransferase-like 3; ACSL4, acyl-CoA synthetase long-chain family member 4; TFRC, transferrin receptor; AIM2, absent in melanoma 2; NFS1, cysteine desulfurase; ZFP64, zinc finger protein 64; HIF-1α, hypoxia-inducible factor 1-alpha; GCLC, glutamate-cysteine ligase catalytic subunit; GPX4, glutathione peroxidase 4; GCH1, GTP cyclohydrolase 1; FTH1, ferritin heavy chain 1; NASH, non-alcoholic steatohepatitis; SI-ALI, sepsis-induced acute lung injury; IVDD, intervertebral disc degeneration; SARDS, sepsis-associated acute respiratory distress syndrome; LC, lung cancer; HCC, hepatocellular carcinoma; TNBC, triple-negative breast cancer; PCa, prostate cancer; CRC, colorectal cancer.

Histone KL-la serves as a critical intermediary linking cellular metabolism with epigenetic regulation, playing a significant role in modulating disease-related ferroptosis through transcriptional reprogramming. This modulation substantially impacts inflammatory pathways and disease outcomes. The regulatory functions of histone KL-la are characterized by context-dependence, wherein it generally enhances ferroptosis and aggravates tissue damage in non-cancerous diseases. Conversely, in malignancies, it inhibits ferroptosis, thereby promoting tumor progression, invasion, and resistance to therapy (**Figure 4**).

#### 4.2.1 Histone KL-la regulation of ferroptosis in non-tumor diseases

In non-tumor diseases, metabolic reprogramming-induced lactate accumulation triggers histone KL-la modifications that coordinately propel ferroptosis through transcriptional reprogramming to simultaneously attenuate antioxidant defenses, disrupt lipid homeostasis, and perturb iron metabolism, thereby aggravating disease progression (24, 25, 81).

Histone KL-la directly targets and undermines cellular antioxidant systems, thereby initiating ferroptosis and inflammatory injury. In models of non-alcoholic steatohepatitis (NASH), lactate-induced H3K18la systematically diminishes hepatocellular antioxidant capacity. This is achieved through the simultaneous upregulation of the transcription factors ATF3, ATF4, and CHAC1, coupled with the downregulation of the essential antioxidant protein GPX4, ultimately leading to ferroptosis and inflammatory damage (25).

Additionally, histone KL-la facilitates the provision of abundant lipid substrates necessary for ferroptotic processes by upregulating the key enzyme ACSL4, either directly or indirectly, thereby exacerbating pathological damage. In models of intervertebral disc degeneration (IVDD) induced by needle puncture, H3K18la directly enhances ACSL4 transcription (24). Conversely, in sepsis-induced lung injury (SILI), H3K18la indirectly increases ACSL4 expression through N6-methyladenosine-dependent upregulation of the methyltransferase METTL3. This series of events leads to the accumulation of lipid peroxidation substrates, which in turn promotes alveolar epithelial ferroptosis (81) and exacerbates both disc degeneration and pulmonary injury. Moreover, histone KL-la contributes to intracellular iron overload by modulating genes involved in iron metabolism. In the context of sepsis-associated acute respiratory distress syndrome (SI-ARDS), the enrichment of H3K14la at the promoters of the transferrin receptor (TFRC) and ferroportin (SLC40A1) leads to an increase in TFRC expression and a concurrent suppression of SLC40A1. This regulatory mechanism results in an elevated net iron influx into pulmonary endothelial cells, thereby inducing ferroptosis and expediting the progression from acute respiratory distress syndrome to respiratory failure (90).

Investigations across NASH, IVDD, SILI, and SI-ARDS models demonstrate that histone KL-la orchestrates a pro-ferroptotic transcriptional network through coordinated regulation of multiple critical targets including GPX4, ACSL4, and TFRC. Distinct KL-la sites exhibit functional preferences, with H3K18la primarily regulating genes associated with stress response and lipid metabolism, whereas H3K14la shows a higher specificity for genes involved in iron transport. The extent to which this site-specific functionality is influenced by local chromatin environments or specialized writer enzymes remains a critical unresolved mechanistic question. Future endeavors should leverage epigenetic editing platforms and multi-omics approaches to precisely dissect the molecular determinants directing site-specific transcriptional programs, while systematically exploring potential functions of additional sites such as H3K9 and H3K27, thereby establishing theoretical foundations for precision therapeutic strategies targeting ferroptosis.

#### 4.2.2 Histone KL-la regulation of ferroptosis in tumor diseases

In tumor microenvironments, the persistent accumulation of lactate driven by the Warburg effect leads to elevated levels of histone KL-la. In stark contrast to non-tumor diseases, this modification is repurposed as a crucial pro-survival signal that coordinately suppresses ferroptosis through multifaceted mechanisms, thereby facilitating tumor growth, metastasis, and therapeutic resistance.

Histone KL-la downregulates critical ferroptotic execution enzymes, thereby limiting the generation of lipid peroxidation substrates. In the context of lung cancer (LC), H3K18la transcriptionally upregulates AIM2, which facilitates the ubiquitination-mediated degradation of STAT5B. This process subsequently suppresses the expression of ACSL4 and reduces the synthesis of polyunsaturated fatty acid phospholipids, ultimately decreasing susceptibility to ferroptosis (21). Additionally, KL-la amplifies antioxidant systems through diverse pathways. In hepatocellular carcinoma, H3K18la concurrently reduces intracellular Fe²^+^ levels via cysteine desulfurase (NFS1) upregulation while augmenting GPX4 expression (22). In prostate cancer, p300-catalyzed lactate-dependent H3K18la formation upregulates HIF-1α to ultimately enhance GPX4 expression (33). In colorectal cancer, H4K12la fortifies glutathione biosynthesis through glutamate-cysteine ligase catalytic subunit (GCLC) upregulation, furnishing abundant GPX4 cofactor to systematically reinforce antioxidant defenses (91). Furthermore, KL-la confers enhanced buffering capacity upon tumor cells by upregulating iron storage proteins and alternative antioxidant pathways. In triple-negative breast cancer(TNBC), H3K18la promotes ferritin heavy chain 1 (FTH1) and GTP cyclohydrolase 1 (GCH1) transcription via transcription factor ZFP64 upregulation. FTH1 augments iron sequestration, while GCH1 provides GPX4-independent antioxidant protection through coenzyme Q10 maintenance, synergistically suppressing ferroptosis and conferring chemoresistance (23).

These studies demonstrate that histone KL-la, and specifically H3K18la, plays a protective role against ferroptosis in tumors, which is in stark contrast to its functions in inflammatory diseases. This context-dependent behavior suggests that the epigenetic and transcriptional landscapes unique to tumors fundamentally reprogram the biological functions of identical modifications. We hypothesize that components of the tumor microenvironment, such as oncogenic signaling and chronic hypoxia, may induce alterations in chromatin architecture, thereby influencing KL-la to preferentially enhance and activate genetic programs associated with cell survival rather than those promoting cell death.

### 4.3 Histone KL-la regulation of NETosis in non-tumor diseases

**Figure 5:**
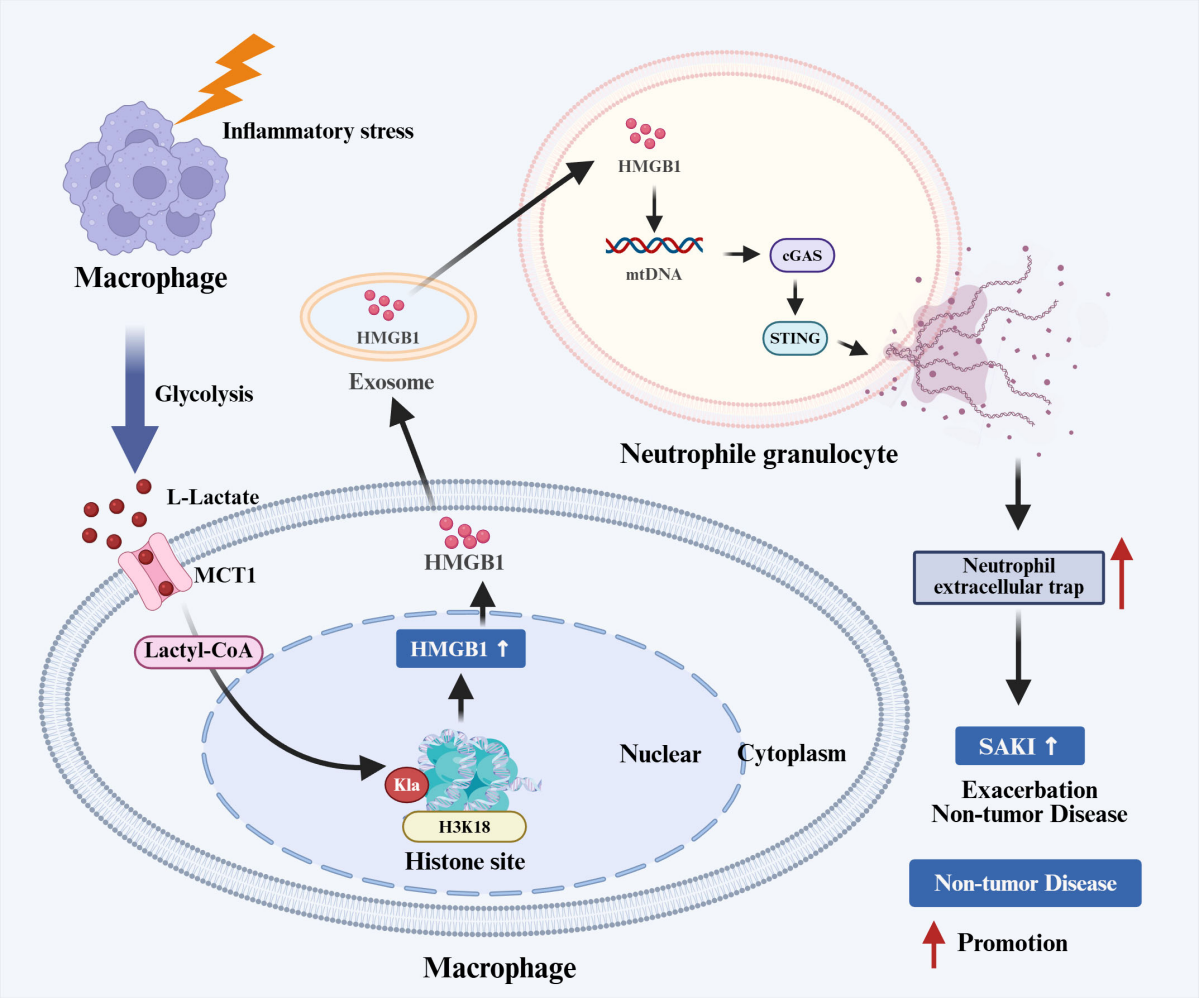
Histone KL-la regulation of NETosis in non-tumor diseases. Inflammatory stress enhances macrophage glycolysis, promoting L-lactate entry via MCT1 and lactyl-CoA generation to induce H3K18la elevation. H3K18la upregulates macrophage HMGB1 expression and facilitates its exosomal release. HMGB1 acts on neutrophils to activate the mtDNA-cGAS-STING pathway, inducing NET release and thereby aggravating SAKI. H3K18la, histone H3 lysine 18 lactylation; MCT1, monocarboxylate transporter 1; lactyl-CoA, lactyl-coenzyme A; HMGB1, high mobility group box 1; mtDNA, mitochondrial DNA; cGAS, cyclic GMP-AMP synthase; STING, stimulator of interferon genes; NET, neutrophil extracellular trap; SAKI, sepsis-associated acute kidney injury.

Current research on the regulation of NETosis by histone KL-la is still in its early stages. Preliminary evidence indicates that in acute systemic inflammatory conditions, KL-la may act as an inflammatory amplifier, promoting NETosis and subsequent tissue damage via intercellular signaling pathways (**Figure 5**).

Using sepsis-associated acute kidney injury (SAKI) as a case study, the research elucidated the role of the “lactate-induced acidification–HMGB1–NETosis” axis. Research indicates that the enhancement of glycolysis in inflammation-driven macrophages results in the accumulation of lactate, which leads to an increase in H3K18la levels. This epigenetic modification specifically upregulates the transcription of HMGB1. Following this, HMGB1 is released from macrophages via exosomes and interacts with neutrophils, triggering NETosis and the release of neutrophil extracellular traps (NETs). Components of NETs, such as neutrophil elastase and citrullinated histone H3, directly damage renal tubular epithelial cells, thereby exacerbating inflammatory infiltration and tissue damage (92).

Critical unresolved questions emerge regarding what determines H3K18la-specific targeting of genes such as HMGB1 within macrophages, whether intrinsic neutrophil KL-la modifications directly regulate core NETotic enzymes such as PAD4, whether this regulatory axis operates universally across severe infection models, and whether functional outcomes vary contingent upon inflammatory contexts or disease stages.

Collectively, preliminary investigations reveal novel mechanisms by which histone KL-la regulates NETosis through intercellular communication networks. Future research efforts must validate this regulatory framework across a wider array of disease models while elucidating the cellular and molecular specificity involved, thereby providing conceptual foundations for immune interventions aimed at addressing pathological NETosis.

## 5 Non-histone KL-la regulation of LCD in diseases

In addition to the transcriptional reprogramming mediated by histone lactylation, lactate directly modifies functional proteins through non-histone lysine lactylation (KL-la), facilitating rapid and precise regulation of cellular signaling. Unlike histone modifications, non-histone KL-la directly influences the stability, activity, subcellular localization, and intermolecular interactions of target proteins at the post-translational level. Accumulating evidence demonstrates that non-histone KL-la orchestrates pivotal yet complex roles in disease progression through regulation of LCD modalities including pyroptosis and ferroptosis, exhibiting profound context-dependence and target specificity. In non-tumor pathologies such as inflammation, ischemia-reperfusion injury, and neurodegenerative disorders, it frequently promotes LCD by stabilizing pro-inflammatory proteins or compromising antioxidant defenses to amplify tissue injury. Conversely, within tumor contexts, identical modifications suppress LCD through mechanisms including antioxidant system enhancement, thereby facilitating tumor survival and therapeutic resistance. This chapter provides a systematic delineation of the molecular mechanisms by which non-histone KL-la regulates pyroptosis and ferroptosis. It constructs an integrative conceptual framework linking modification, target, function, and disease through a comparative analysis of tumor and non-tumor action modalities. Furthermore, it examines the molecular determinants that underlie site-specificity and context-dependence, thereby establishing theoretical foundations for the development of disease-selective precision therapeutics (**Table 2**).

**Table 2:** Non-histone K_L-la_-mediated regulation of LCD in diseases.

Non-histone lactylation site		Downstream target	Effect on LCD		Disease	Effect on disease	Reference
NEDD4	K33	Caspase-11	Pyroptosis	↑	AILI(Liver)	Exacerbation	(76)
SOX10	–	GSDMD			AS		(77)
NLRP3	K166	ASC			SLE		(94)
CNPY3	–	CatB		↓	PCa		(95)
PKM2	K433	P50			BCa		(75)
METTL3	–	TFRC	Ferroptosis	↑	ICH	Exacerbation	(96)
tau	K677	NCOA4			AD		(97)
PCK2	K100	OXSM			HIRI		(46)
MDH2	K241	GPX4			MIRI		(98)
NSUN2	K508	GCLG		↓	GC		(99)
HDAC1	K412	H3K27			CRC		(100)
LSD1	K503	TFRC			Melanoma		(101)

NEDD4, neural precursor cell expressed developmentally down-regulated protein 4; K33, lysine 33; Caspase-11, cysteine-aspartic acid protease 11; SOX10, SRY-box transcription factor 10; GSDMD, gasdermin D; NLRP3, NOD-like receptor family pyrin domain-containing 3; K166, lysine 166; ASC, apoptosis-associated speck-like protein containing a CARD; CNPY3, canopy FGF signaling regulator 3; CatB, cathepsin B; PKM2, pyruvate kinase M2; K433, lysine 433; p50, NF-κB p50 subunit; METTL3, methyltransferase-like 3; TFRC, transferrin receptor; tau, microtubule-associated protein tau; K677, lysine 677; NCOA4, nuclear receptor coactivator 4; PCK2, phosphoenolpyruvate carboxykinase 2 (mitochondrial); K100, lysine 100; OXSM, 3-oxoacyl-ACP synthase (mitochondrial); MDH2, malate dehydrogenase 2; K241, lysine 241; GPX4, glutathione peroxidase 4; NSUN2, NOP2/Sun RNA methyltransferase 2; K508, lysine 508; GCLG, glutamate-cysteine ligase modifier subunit; HDAC1, histone deacetylase 1; K412, lysine 412; H3K27, histone H3 lysine 27; LSD1, lysine-specific demethylase 1; K503, lysine 503; AILI, acute inflammatory liver injury; AS, ankylosing spondylitis; SLE, systemic lupus erythematosus; PCa, prostate cancer; BCa, breast cancer; ICH, intracerebral haemorrhage; AD, Alzheimer’s disease; HIRI, hepatic ischemia–reperfusion injury; MIRI, myocardial ischemia–reperfusion injury; GC, gastric cancer; CRC, colorectal cancer.

### 5.1 Non-histone KL-la regulation of pyroptosis in diseases

The non-histone protein KL-la plays a crucial role as a lactate-mediated post-translational modification (PTM) that facilitates the swift and direct regulation of protein functions, thereby linking cellular metabolism with pyroptotic signaling pathways. Research indicates that KL-la exhibits complex and context-dependent regulatory functions in the modulation of pyroptosis. In non-tumor diseases, KL-la primarily induces pyroptosis by stabilizing inflammatory caspases and promoting the assembly of inflammasomes, which in turn exacerbates tissue damage.

**Figure 6:**
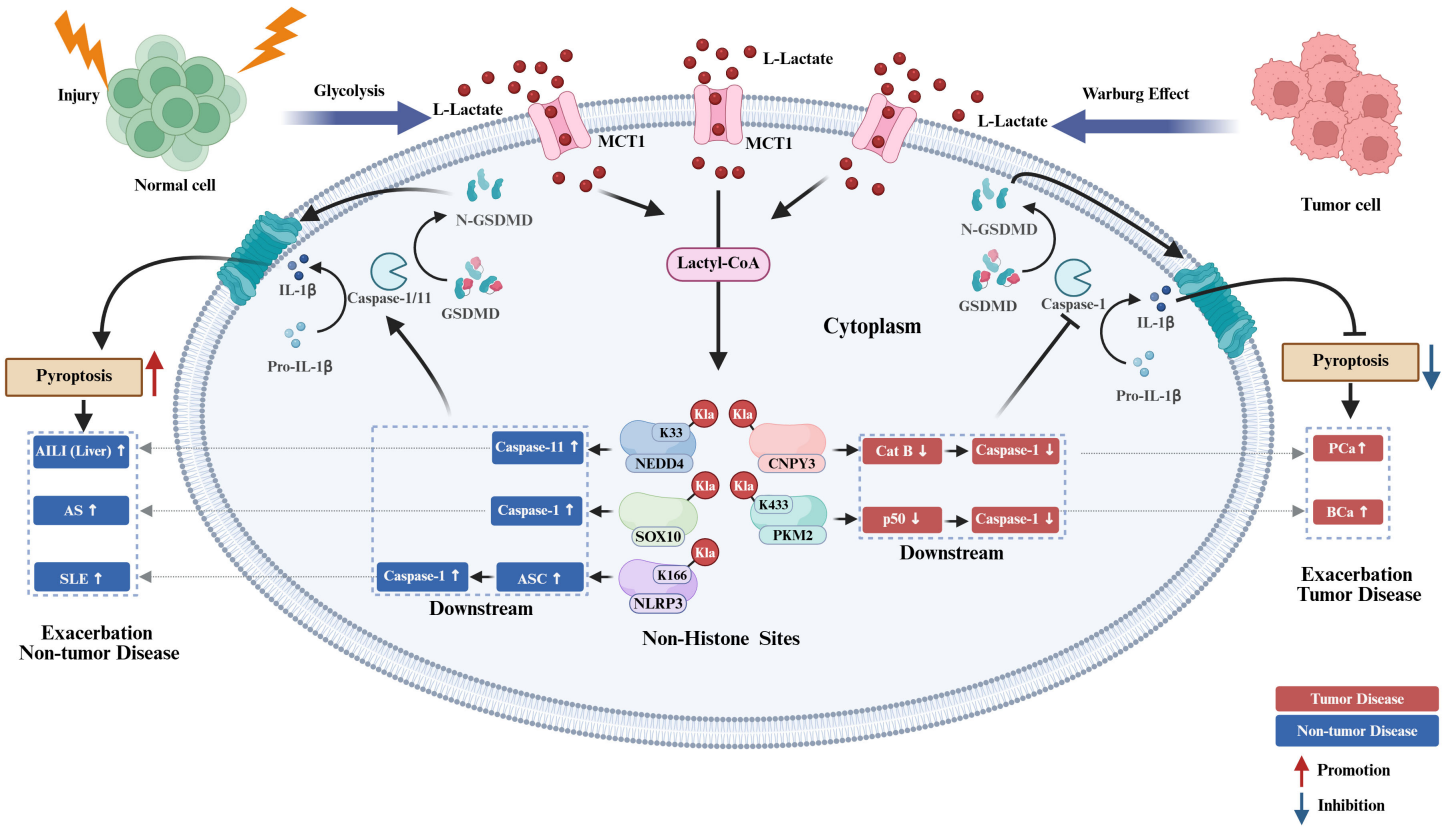
Non-histone KL-la regulation of pyroptosis in diseases. Under conditions of inflammation, injury, or tumor-associated metabolic reprogramming, glycolytic enhancement drives L-lactate accumulation that undergoes MCT1-mediated intracellular transport and lactyl-CoA conversion. Lactyl-CoA serves as a donor mediating KL-la at histone sites including H3K18, H3K14, and H4K12, alongside non-histone sites including NEDD4, SOX10, NLRP3, CNPY3, and PKM2, to regulate downstream inflammasome, caspase, iron homeostasis, and lipid peroxidation-associated pathways. This process can either promote or suppress pyroptosis and ferroptosis in non-tumor diseases to aggravate tissue injury, whereas within tumor cells, Warburg effect-driven lactate signaling reshapes cell death susceptibility through KL-la to influence tumor progression. Red arrows denote promotional effects while blue arrows indicate inhibitory actions. MCT1, monocarboxylate transporter 1; lactyl-CoA, lactyl-coenzyme A; KL-la, lysine lactylation; H3K18, histone H3 lysine 18; H3K14, histone H3 lysine 14; H4K12, histone H4 lysine 12; NEDD4, neural precursor cell expressed developmentally down-regulated protein 4; SOX10, SRY-box transcription factor 10; NLRP3, NOD-like receptor family pyrin domain containing 3; CNPY3, canopy fibroblast growth factor signaling regulator 3; PKM2, pyruvate kinase M2.

Conversely, within tumor microenvironments, similar modifications inhibit pyroptosis by maintaining lysosomal homeostasis or interfering with competitive metabolic enzymes, thereby promoting immune evasion and tumor progression. This bidirectional regulatory capacity indicates that metabolic reprogramming significantly influences cellular fate determination through distinct patterns of non-histone KL-la modifications. This chapter systematically elucidates the specific mechanisms underlying the regulation of pyroptosis by non-histone KL-la in both tumor and non-tumor diseases. Furthermore, through a comparative mechanistic analysis, it explores the biological foundations of functional reversal, providing conceptual frameworks for context-dependent therapeutic targeting strategies (**Figure 6**).

#### 5.1.1 Non-histone KL-la regulation of pyroptosis in non-tumor diseases

In the context of non-tumor diseases, the accumulation of lactate due to metabolic or inflammatory stress induces the non-histone modification KL-la, which modulates the inflammasome-Caspase-Gasdermin axis at the post-translational level, thereby promoting cellular pyroptosis and exacerbating disease progression. The regulatory mechanisms of this process encompass three principal aspects. First, KL-la enhances pyroptotic signaling by stabilizing caspase proteins. In the context of acetaminophen-induced liver injury (AILI), the enhancement of glycolysis in macrophages results in the accumulation of lactate, which facilitates the lactylation of E3 ubiquitin ligase NEDD4 at lysine 33 (NEDD4-K33la) (76). This post-translational modification inhibits NEDD4-mediated ubiquitination and subsequent degradation of caspase-11, leading to an abnormal accumulation of caspase-11. This accumulation triggers non-canonical pyroptosis, thereby exacerbating hepatic inflammation and injury. Second, KL-la enhances the expression of key pyroptotic genes by modifying transcription factors. In models of atherosclerosis (AS), tumor necrosis factor-alpha (TNF-α) induces lactate production in vascular smooth muscle cells (VSMCs), leading to the lactylation of the transcription factor SOX10. This modification increases SOX10’s transcriptional activity, resulting in the upregulation of GSDMD and caspase-1 expression. Consequently, this process promotes VSMC pyroptosis and accelerates plaque progression (77). Furthermore, KL-la directly modifies core inflammasome components to facilitate assembly. In systemic lupus erythematosus (SLE)-associated pregnancy complications, elevated trophoblast lactate modifies NLRP3 at lysine 166 (NLRP3-K166la), strengthening its interaction with adaptor protein ASC to promote NLRP3 inflammasome assembly, caspase-1 activation, and consequent pyroptosis that impairs placental function (94).

In non-tumor diseases, non-histone KL-la enhances pyroptosis and inflammation by stabilizing caspases, promoting inflammasome assembly, and boosting pyroptotic gene transcription. However, research has mostly addressed indirect or singular pathways, lacking detailed insight into site-specific, temporal, and context-dependent mechanisms. Future studies should thoroughly explore lactylation enzyme dynamics and non-histone modifications to develop precise intervention strategies.

#### 5.1.2 Non-histone KL-la regulation of pyroptosis in tumor diseases

In tumor microenvironments characterized by sustained elevated lactate levels, the non-histone protein KL-la demonstrates pyroptosis-inhibitory effects, which represent strategies employed by tumor cells to resist cell death and adapt for survival. Mechanistically, this inhibition is achieved through modulation at key nodes within the pyroptotic signaling pathways. KL-la attenuates inflammasome activation by maintaining lysosomal homeostasis. In prostate cancer (PCa), the lactylation of the molecular chaperone CNPY3, induced by lactate, enhances the integrity of the lysosomal membrane and reduces the leakage of cathepsin B. This process inhibits the activation of the NLRP3/caspase-1/GSDMD pathway, thereby suppressing pyroptosis and facilitating tumor growth (95). Furthermore, KL-la disrupts pyroptotic signal transduction by competitively modifying metabolic enzymes. In bladder cancer (BCa), the lactylation of pyruvate kinase M2 (PKM2) at lysine 166 (PKM2-K166la) competitively inhibits acetylation at this site, leading to a reduction in the activation of the NLRP1/caspase-1/GSDMD pathway. This attenuation suppresses pyroptosis and diminishes anti-tumor immunity (75).

In conclusion, non-histone KL-la primarily demonstrates pyroptosis-suppressive functions in tumor environments, in stark contrast to its pro-pyroptotic effects observed in non-tumor pathologies, thereby highlighting the significant context-dependent condition of its functional roles. Current research in this field is still in its early stages, requiring further exploration across a broader range of tumor types. Future studies should aim to elucidate the mechanisms by which tumor cells achieve substrate- and site-specific modification precision. Additionally, investigations should focus on the lactate-dependent dynamic regulation of modifying enzyme activities and systematically examine the roles of KL-la within tumor immune microenvironments. These efforts are essential for establishing the theoretical foundations necessary for developing anti-tumor therapies based on the reversal of lactylation and the restoration of pyroptosis.

### 5.2 Non-histone KL-la regulation of ferroptosis in diseases

**Figure 7:**
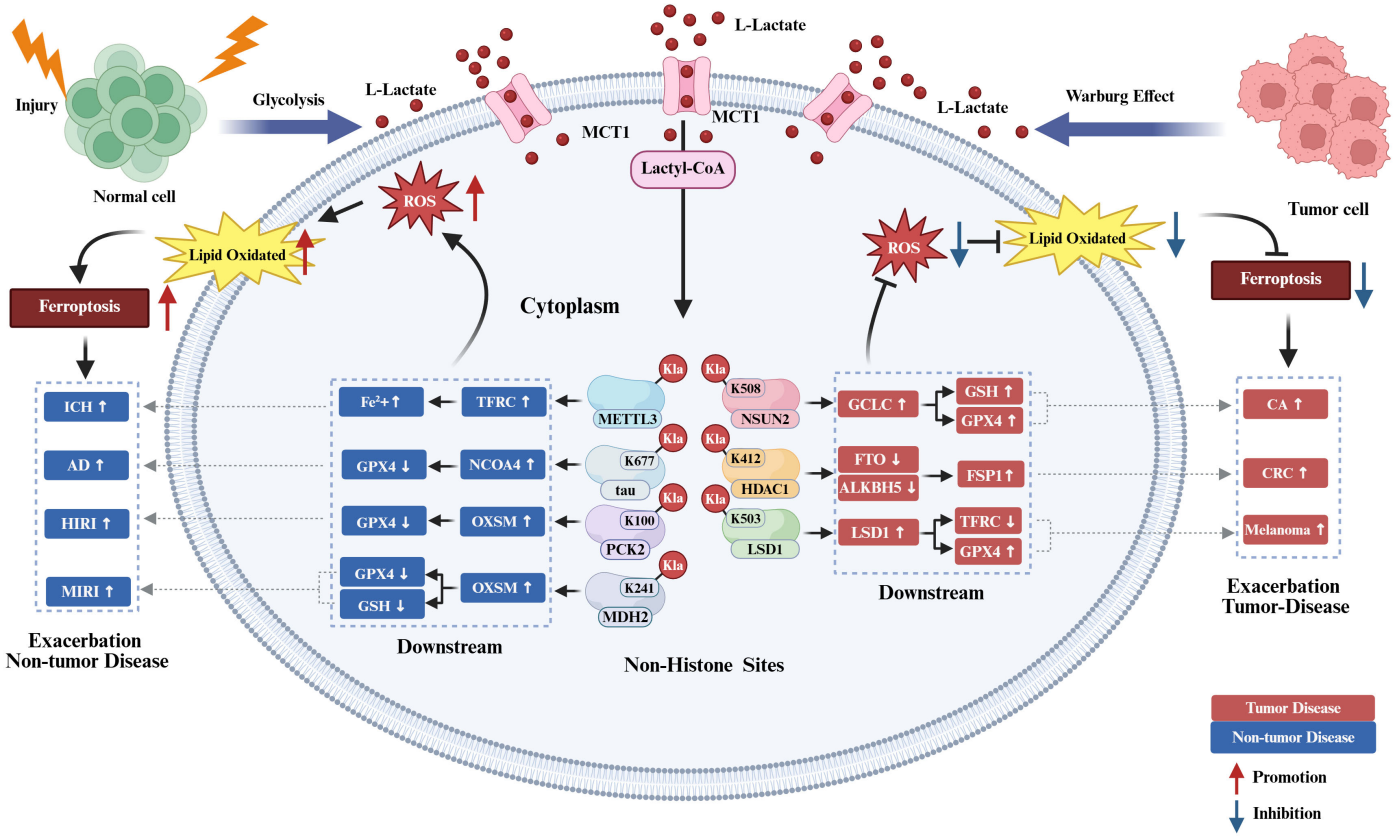
Non-histone KL-la regulation of ferroptosis in diseases. In non-tumor diseases, injury and stress induce enhanced cellular glycolysis, promoting L-lactate transport via MCT1 and lactyl-CoA conversion to mediate KL-la modifications at multiple non-histone sites including METTL3, tau, PCK2, MDH2, and NSUN2. Through regulation of iron homeostasis, lipid peroxidation, and antioxidant systems, these modifications promote ferroptosis and aggravate disease progression in ICH, AD, HIRI, and MIRI. Within tumors, the Warburg effect drives persistent lactate accumulation, enabling non-histone KL-la to reshape antioxidant and iron metabolism pathways through targets including GCLC, GPX4, FSP1, and TFRC, thereby suppressing lipid peroxidation and ferroptosis to promote progression of tumors including LC, CRC, and melanoma. Red and blue colors represent tumor and non-tumor diseases, respectively; red upward arrows indicate promotion while blue downward arrows indicate inhibition. MCT1, monocarboxylate transporter 1; lactyl-CoA, lactyl-coenzyme A; KL-la, lysine lactylation; METTL3, methyltransferase-like 3; tau, microtubule-associated protein tau; PCK2, phosphoenolpyruvate carboxykinase 2; MDH2, malate dehydrogenase 2; NSUN2, NOP2/Sun RNA methyltransferase 2; GCLC, glutamate-cysteine ligase catalytic subunit; GPX4, glutathione peroxidase 4; FSP1, ferroptosis suppressor protein 1; TFRC, transferrin receptor; ICH, intracerebral hemorrhage; AD, Alzheimer’s disease; HIRI, hepatic ischemia-reperfusion injury; MIRI, myocardial ischemia-reperfusion injury; LC, lung cancer; CRC, colorectal cancer.

Non-histone KL-la represents a crucial lactate-mediated post-translational modification (PTM) that exhibits significant context-dependence in the regulation of ferroptosis. In non-tumor diseases, non-histone KL-la promotes ferroptosis by disrupting iron homeostasis and inhibiting GPX4, thereby exacerbating tissue injury. Conversely, within tumor microenvironments, non-histone KL-la inhibits ferroptosis by enhancing antioxidant defenses and limiting iron uptake, thus facilitating tumor cell resistance to ferroptosis, survival, and progression. This dual regulatory capacity highlights the disease context-dependent functionality of non-histone KL-la. This chapter provides a systematic examination of the roles and mechanisms of non-histone KL-la in ferroptosis regulation across various diseases, while exploring the biological underpinnings of its context-dependent functionality (**Figure 7**).

#### 5.2.1 Non-histone KL-la regulation of ferroptosis in non-tumor diseases

Under conditions of ischemic and inflammatory stress, the enhancement of glycolysis and subsequent accumulation of lactate lead to the induction of non-histone KL-la. This induction disrupts iron homeostasis and antioxidant defenses at the post-translational level, thereby promoting ferroptosis and contributing to disease progression.

The non-histone protein KL-la disrupts intracellular iron homeostasis by upregulating iron uptake proteins. In models of intracerebral hemorrhage, lactate modifies and activates METTL3, leading to an increased expression of transferrin receptor 1 (TFRC), which results in neuronal iron overload, ferroptosis, and exacerbated cerebral injury (96). Furthermore, KL-la induces uncontrolled lipid peroxidation by inhibiting the GPX4 core antioxidant system. In Alzheimer’s disease (AD), lactylation of the tau protein at lysine 677 activates the p38 MAPK-NCOA4 pathway, resulting in the downregulation of GPX4 and the induction of microglial ferroptosis (97). In the hepatic ischemia-reperfusion injury (HIRI), lactylation of phosphoenolpyruvate carboxykinase 2 (PCK2) at lysine 100 leads to the upregulation of OXSM and the suppression of GPX4 (46). Similarly, in myocardial ischemia-reperfusion injury (MIRI), lactylation of malate dehydrogenase 2 (MDH2) at lysine 241 promotes cardiomyocyte ferroptosis through the inhibition of GPX4 and depletion of glutathione (98).

In conclusion, these researches indicates that in non-tumor diseases, non-histone lactylation facilitates ferroptosis through two primary pathways: the disruption of iron homeostasis and the inhibition of GPX4, which collectively exacerbate tissue damage and disease progression. However, significant knowledge gaps remain, particularly regarding the regulation of lactylation on the key ferroptotic driver ACSL4 and its pathological consequences.

#### 5.2.2 Non-histone KL-la regulation of ferroptosis in tumor diseases

Unlike the pro-ferroptotic effects seen in non-tumor conditions, non-histone KL-la mainly suppresses ferroptosis in lactate-rich tumor environments. This may be an adaptive mechanism that helps tumor cells survive metabolic stress. It achieves this by boosting antioxidant capacity and limiting iron uptake.

KL-la enhances the antioxidant defense mechanisms of tumor cells through both canonical GPX4-dependent pathways and non-canonical alternative pathways. In gastric cancer(GC), lactate-induced RNA methyltransferase NSUN2 lactylation at lysine 508 (NSUN2-K508la) augments glutathione synthesis and GPX4 activity through upregulation of the glutathione synthesis rate-limiting enzyme glutamate-cysteine ligase catalytic subunit (GCLC), thereby efficiently eliminating lipid peroxides (99). In colorectal cancer(CRC), histone deacetylase 1 (HDAC1) lactylation at lysine 412 (HDAC1-K412la) upregulates ferroptosis suppressor protein 1 (FSP1) expression via epigenetic modulation to activate the FSP1-ubiquinol (CoQH_2_) non-canonical antioxidant axis for synergistic ferroptosis suppression (100). Furthermore, KL-la diminishes ferroptotic susceptibility through iron uptake inhibition. In melanoma, lactylated lysine-specific demethylase 1 (LSD1) at lysine 508 (LSD1-K508la) complexes with FosL1 to directly repress TFRC transcription and limit iron influx, while concurrently stabilizing GPX4 and SLC7A11 proteins to multilayered ferroptosis blockade and tumor growth promotion (101).

In summary, Tumor cells use non-histone KL-la as a key regulator to suppress ferroptosis and ensure survival, showing effects opposite to those in non-tumor diseases and highlighting the importance of context. However, significant gaps in knowledge persist, such as the unexplored lactylation regulation of ACSL4. Additionally, it is unclear if common upstream targets or pathways control non-histone KL-la modifications across different tumor models. Systematic investigation is needed to fully understand tumor metabolic adaptation and develop targeted therapies.

## 6 Therapeutic strategies targeting lactate metabolism and KL-la enzymes to regulate LCD

Lactate is not simply the end product of glycolysis; it plays a crucial role in regulating LCD, such as ferroptosis and pyroptosis, through the induction of protein lactylation modifications at both epigenetic and post-translational levels. As a result, targeting lactate metabolism and the enzymes involved in its modification has become an important therapeutic strategy.

This strategy operates on two levels: inhibiting lactate production to affect lactylation at its source, and directly targeting lactylation enzymes to regulate protein modification and correct LCD processes. This chapter reviews recent progress, first summarizing chemical drugs, traditional Chinese medicine, and gene therapy for reducing lactate production, then detailing interventions using chemical drugs, traditional Chinese medicine, and small-molecule drugs on lactylation enzymes. We analyzes the mechanisms and therapeutic potential in diseases, laying the groundwork for new treatments based on the lactate-lactylation axis (**Table 3**).

**Table 3:** Therapeutic strategies targeting lactate metabolism and K_L-la_–related enzymes to regulate lytic cell death.

Disease model	Drug name	Drug category	Molecular target	Effect on LCD		Effect on disease	Reference
MIRI	Dex	CD	NR3C1	Ferroptosis	↓	Inhibition	(98)
BCa	Mannose		PKM2	Pyroptosis	↑	Inhibition	(75)
LC	Shikonin	TCM	PKM2	Ferroptosis	↑	Inhibition	(21)
PCa	Evodiamine	TCM	PKM2	Ferroptosis	↑	Inhibition	(33)
AD	si-LDHA	GT	LDHA	Pyroptosis	↓	Inhibition	(74)
IVDD	AAV9-si-Ldha	GT	LDHA	Ferroptosis	↓	Inhibition	(24)
CRC	SAHA/TSA	CD	HDAC1	Ferroptosis	↑	Inhibition	(100)
PCa	Gambogic acid	TCM	SIRT1	Pyroptosis	↑	Inhibition	(95)
NASH	Tectorigenin	TCM	HDAC1	Ferroptosis	↓	Inhibition	(25)
HIRI	KAT8-IN-1	SMD	PCK2	Ferroptosis	↓	Inhibition	(46)

MIRI, myocardial ischemia–reperfusion injury; BCa, bladder cancer; LC, lung cancer; PCa, prostate cancer; CRC, colorectal cancer; AD, Alzheimer’s disease; IVDD, intervertebral disc degeneration; NASH, nonalcoholic steatohepatitis; HIRI, hepatic ischemia–reperfusion injury; Dex, dexamethasone; Mannose, mannose; CD, chemical drug; TCM, traditional Chinese medicine; GT, gene therapy; SMD, small-molecule drug; NR3C1, nuclear receptor subfamily 3 group C member 1 (glucocorticoid receptor); PKM2, pyruvate kinase M2; LDHA, lactate dehydrogenase A; HDAC1, histone deacetylase 1; SIRT1, sirtuin 1; PCK2, phosphoenolpyruvate carboxykinase 2 (mitochondrial); SAHA, suberoylanilide hydroxamic acid; TSA, trichostatin A; AAV9, adeno-associated virus serotype 9; si-LDHA, small interfering RNA targeting LDHA; KAT8-IN-1, lysine acetyltransferase 8 inhibitor 1.

### 6.1 Therapeutic strategies targeting L-lactate generation inhibition to regulate LCD

A primary strategy for modulating the lactate-lactylation axis in LCD intervention entails the suppression of L-lactate production at its metabolic sources. By reducing lactate levels, either directly or indirectly, lactate-induced lactylation modifications are attenuated, thereby reprogramming dysregulated LCD processes to provide targets for disease intervention. This subsection provides a systematic review of three strategic categories based on this rationale: chemical drugs (CD), traditional Chinese medicine (TCM), and gene therapy (GT). Although these strategies differ mechanistically, they share a fundamental operational principle: the reduction of pathological lactate accumulation through the targeting of glycolytic molecules such as pyruvate kinase M2 (PKM2), pyruvate dehydrogenase kinase 4 (PDK4), and lactate dehydrogenase A (LDHA). This targeting modulates downstream KL-la and LCD pathways, including ferroptosis and pyroptosis, ultimately aiming to alleviate tissue injury or inhibit tumor progression. The subsequent sections systematically elucidate the molecular mechanisms, application paradigms, and research limitations associated with each strategic category.

#### 6.1.1 CD strategies targeting L-lactate generation inhibition to regulate LCD

Chemical drugs regulate LCD processes to ameliorate disease prognoses through lactate synthesis inhibition that reduces intracellular lactate concentrations and consequent KL-la attenuation. In non-tumor pathologies, myocardial ischemia-reperfusion injury (MIRI) exemplifies wherein dexmedetomidine promotes nuclear receptor subfamily 3 group C member 1 (NR3C1) phosphorylation to downregulate PDK4 expression and diminish lactate generation. Resultant lactate level reduction inhibits malate dehydrogenase 2 (MDH2) lactylation modification, subsequently upregulating GPX4 expression to alleviate cardiomyocyte ferroptosis and ultimately mitigate myocardial injury (98). Within tumor contexts, Mannose suppresses glycolysis through PKM2 binding to reduce bladder cancer(BCa) cellular lactate concentrations. This mechanism inhibits PKM2 lactylation while promoting acetylation modification to activate nuclear factor kappa B (NF-κB) signaling, inducing tumor cell pyroptosis and suppressing tumor progression (75).

Current CD strategies mainly focus on two key glycolytic points: PKM2 and PDK4. However, other glycolytic components like LDHA, PFKFB3, and MCT4 could also be targeted to inhibit lactate production, though they are underdeveloped pharmaceutically (102). Future research should broaden target options to improve strategy effectiveness.

#### 6.1.2 TCM strategies targeting L-lactate generation inhibition to regulate LCD

Traditional Chinese medicine(TCM) bioactive constituents constitute alternative pathways for lactate generation modulation affecting LCD. Recent evidence indicates that select Chinese medicine monomers reduce tumor cellular lactate synthesis through glycolytic enzyme targeting, thereby downregulating pro-survival protein lactylation modifications to ultimately induce tumor cell LCD.

In lung cancer(LC), shikonin substantially diminishes cellular lactate concentrations through pyruvate kinase M2 (PKM2) activity inhibition. This attenuates absent in melanoma 2 (AIM2) inflammasome receptor lactylation modification, subsequently reducing ubiquitination-mediated signal transducer and activator of transcription 5B (STAT5B) degradation to upregulate ACSL4 expression, ultimately triggering lung cancer cell ferroptosis and suppressing tumor growth (21). In prostate cancer(PCa), evodiamine similarly reduces lactate generation through PKM2 activity inhibition. Consequent lactate level reduction attenuates hypoxia-inducible factor 1-alpha (HIF-1α) lactylation modification to downregulate downstream target gene GPX4 expression, enhancing intracellular lipid peroxidation to induce prostate cancer cell ferroptosis and thereby suppressing proliferative, invasive, and metastatic capacities (33).

Nevertheless, field investigations remain constrained. Current TCM strategies predominantly concentrate on lung and prostate malignancies with singular PKM2 targeting. Whether such approaches prove effective across additional lactate-KL-la-LCD axis-regulated tumors including gastric and colorectal cancers remains unresolved. Moreover, therapeutic potentials within non-tumor pathologies such as non-alcoholic steatohepatitis and intervertebral disc degeneration await exploration.

#### 6.1.3 GT strategies targeting L-lactate generation inhibition to regulate LCD

Recent gene therapy(GT) technological advances have enabled intervention strategies targeting L-lactate generation gene suppression that reduce pathological microenvironmental L-lactate concentrations through lactate metabolism enzyme expression interference, inhibit KL-la, thereby regulate LCD occurrence, and improve disease outcomes.

In Alzheimer’s disease investigations, lactate LDHA small interfering RNA application effectively downregulates LDHA protein expression to diminish microglial lactate accumulation. Resultant lactate level reduction inhibits NIMA-related kinase 7 (NEK7) lactylation modification, thereby blocking NEK7-mediated microglial pyroptosis to reduce neuroinflammation and ameliorate pathological manifestations (74). In intervertebral disc degeneration models, adeno-associated virus serotype 9 vector-mediated delivery of LDHA-targeting small interfering RNA silences ldha gene expression to substantially reduce nucleus pulposus cellular lactate concentrations. Lactate generation inhibition attenuates subsequent lactylation modification-driven ferroptotic signaling to protect nucleus pulposus cells and delay intervertebral disc degeneration progression (24).

Current strategic investigations predominantly focus on LDHA targeting within non-tumor pathologies. Future research necessitates target expansion encompassing alternative glycolytic pathway genes including PKM2 or PKM4. Concurrently, validating whether such gene intervention strategies effectively regulate LCD and improve prognoses within tumor contexts constitutes an urgent exploratory direction.

### 6.2 Therapeutic strategies targeting KL-la enzymes to regulate LCD

In addition to suppressing lactate production, targeting enzymes involved in direct lactylation modification offers a crucial alternative strategy for regulating LCD pathways. The levels of lactylation modifications are subject to bidirectional regulation by lactyl transferases and delactylases. Intervening in these enzymatic processes can modulate the states of substrate modification, thereby influencing the occurrence and progression of LCD and providing disease-specific therapeutic opportunities. This section elucidates the strategies for targeting delactylases and lactyl transferases, systematically assessing their therapeutic potential while also identifying current limitations in the field and suggesting future research directions.

#### 6.2.1 CD and TCM strategies targeting delactylases to regulate LCD

Delactylases sustain lactylation modification dynamic equilibrium through lactyl group removal from substrate proteins. Activity modulation of these enzymes directly impacts the KL-la axis, providing disease therapeutic entry points. Contemporary chemical pharmaceuticals and traditional Chinese medicine constituents demonstrate definitive regulatory effects within this domain.

Among chemical drugs, vorinostat (SAHA) and trichostatin A (TSA) suppress histone deacetylase 1 (HDAC1) lactylation while promoting acetylation to upregulate RNA demethylases fat mass and obesity-associated protein (FTO) and alkB homolog 5 (ALKBH5) expression. These demethylases enhance lipid peroxidation and induce colorectal cancer cell ferroptosis through ferroptosis suppressor protein 1 (FSP1) downregulation, suppressing tumor progression (100). Within traditional Chinese medicine constituents, gambogic acid inhibits Sirtuin 1 (SIRT1)-mediated canopy fibroblast growth factor signaling regulator 3 (CNPY3) delactylation. This process disrupts lysosomal membrane integrity, precipitating cathepsin B (CatB) liberation to activate the NLRP3-caspase-1 pathway and induce prostate cancer cell pyroptosis (95). Furthermore, Tectorigenin alleviates hepatocellular injury and fibrosis in non-alcoholic steatohepatitis through hepatocyte HDAC1 expression upregulation that inhibits ferroptosis-related stress gene lactylation including activating transcription factor 3 (ATF3), ATF4, and glutathione-specific gamma-glutamylcyclotransferase 1 (CHAC1) (25).

These therapeutic strategies primarily concentrate on dual targets, specifically HDAC1 and SIRT1, which have shown efficacy in the treatment of colorectal cancer, prostate cancer, and non-alcoholic steatohepatitis. However, the delactylase family comprises several additional members. It remains to be systematically investigated whether alternative members, such as HDAC2/3 and SIRT2/3, represent viable therapeutic targets and whether the applicability of these strategies extends to other pathologies regulated by the KL-la-LCD axis.

#### 6.2.2 SMD strategies targeting lactyl transferases to regulate LCD

Lactyl transferases catalyze lactyl group covalent transfer onto target proteins, constituting pivotal enzymatic mediators of lactylation modifications. Consequently, small molecule drug inhibitor development for specific activity suppression effectively intervenes in the KL-la-LCD axis, furnishing precision disease therapeutic tools. Current investigations demonstrate strategic potential within non-tumor pathologies. In hepatic ischemia-reperfusion injury models, the small molecule inhibitor KAT8-IN-1 substantially reduces mitochondrial phosphoenolpyruvate carboxykinase 2 (PCK2) lactylation concentrations through specific lysine acetyltransferase 8 (KAT8) activity inhibition. Diminished PCK2 modification attenuates its metabolic functionality while promoting Parkin-mediated 3-oxoacyl-acyl carrier protein synthase (OXSM) degradation, thereby suppressing lipid peroxidation pathway activation to ultimately alleviate hepatocellular ferroptosis and hepatic injury (46).

Field investigations are still in early stages, primarily focusing on non-tumor pathologies and ferroptosis, with limited target selection centered on KAT8. Future research should broaden to include small molecule inhibitors for other lactyl transferases like KAT2, HAT1, or KAT5. Additionally, systematic validation of LCD regulatory capacity and its potential to suppress tumor progression across various models is needed.

## 7 Conclusions and future perspectives

Lactate, as a pivotal glycolytic metabolite, has transcended conventional energetic functions to emerge as a critical metabolic signaling molecule. Through mediating histone and non-histone lactylation modifications, it orchestrates a sophisticated regulatory network governing LCD at epigenetic and post-translational tiers. This review systematically elucidates the comprehensive axis spanning lactate metabolism to KL-la modification-mediated LCD regulation and disease progression, while summarizing therapeutic strategies targeting lactate generation suppression or lactylation enzyme modulation. Core findings reveal three fundamental principles: Firstly, KL-la achieves bidirectional regulation of LCD pivotal molecules including GSDMD, GPX4, and NLRP3 at transcriptional and post-translational echelons. Secondly, KL-la-mediated LCD regulatory effects exhibit pronounced context-dependence, manifesting frequently opposing functionalities across tumor versus non-tumor pathologies as jointly determined by disease-specific metabolic microenvironments, inflammatory states, and cellular identities. Thirdly, interventional strategies that target metabolic origins or modification switches provide novel therapeutic pathways for improving disease prognosis through the reprogramming of LCD.

Although KL-la-mediated subtype-specific LCD regulation has been demonstrated, there remain significant gaps in our understanding of the precise molecular networks and dynamic regulatory paradigms involved. In the context of pyroptosis, KL-la functionality has been established in relation to histone modifications, such as H3K18la and H4K12la, as well as non-histone substrates like NLRP3 and caspases. However, critical uncertainties persist regarding its dynamic functional contributions across various pyroptotic stages, including inflammasome priming and GSDMD cleavage. Additionally, the involvement of alternative histone sites, such as H3K14 and H3K9, and the spatiotemporal synergistic interactions between histone and non-histone modification cascades remain to be elucidated. In ferroptosis, KL-la governs cellular fate through core protein regulation including FTH1, GPX4, and ACSL4, yet pivotal questions persist regarding additional histone lactylation site functionalities, mechanistic determinants underlying H3K18la bidirectional GPX4 regulation, direct SLC7A11 and FSP1 modification status, and temporal regulation by substrate-specific writer or eraser enzymes. Within NETosis, preliminary evidence implicates histone KL-la including H3K18la in chromatin structural alterations facilitating NET formation, though additional site-specific modifications alongside direct lactylation-mediated regulation of core executors including peptidylarginine deiminase 4 (PAD4) and neutrophil elastase (NE) remain unexplored. Collectively, as a pivotal nexus bridging metabolic stress and cell death determination, comprehensive dynamic regulatory landscapes governing KL-la functions across LCD modalities necessitate systematic investigation.

The context-dependent nature of histone or non-histone KL-la in LCD-mediated disease pathogenesis manifests across macroscopic disease contexts and microscopic pathological states. Regulatory directionality exhibits profound disease background dependency wherein identical modifications including H3K18la and H4K12la typically promote LCD to exacerbate injury within non-tumor pathologies such as tissue damage, yet suppress LCD to facilitate cellular survival, invasion, and therapeutic resistance within tumor contexts such as lung cancer. This bidirectional regulatory paradigm originates from three fundamental divergences encompassing metabolic characteristics wherein non-tumor disease lactate elevation represents stress-induced, fluctuating patterns triggering acute responses, contrasting sharply with tumor Warburg effect-driven persistent lactate-enriched microenvironments providing stable modification substrates; inflammatory profiles wherein non-tumor pathologies manifest acute pro-inflammatory states aimed at damage clearance yet prone to tissue destruction amplification, whereas tumors exhibit chronic immunosuppressive inflammation facilitating immune evasion; and cellular response orientations wherein the former directs toward clearance and repair while the latter biases toward survival and adaptation. Functional complexity intensifies as even within identical disease contexts, KL-la functionality depends on specific pathological states. Within IBD, H3K18la-mediated pyroptosis suppression yields opposing pathological outcomes in macrophages operating under immune surveillance imperatives in glycogen storage disease-associated IBD versus inflammatory destruction predominance in dextran sulfate sodium-induced ulcerative colitis, underscoring modification functionality determination by cellular functional states within specific microenvironmental niches. Furthermore, the lactylation-LCD-disease regulatory axis exhibits pronounced cell type dependency wherein identical modifications such as H3K18la generate divergent or opposing biological effects across distinct cellular populations within disease categories. Within neurological pathologies, neuronal H3K18la drives pyroptosis through HMGB1 upregulation to intensify injury, whereas microglial H3K18la potentially exerts pyroptosis-suppressive neuroprotective functions through YTHDF3-PRDX3 axis maintenance, revealing KL-la functional plasticity necessitating systematic dissection of cell-intrinsic determinants governing functional heterogeneity. Additionally, the axis manifests target protein dependency wherein biological outcomes critically depend on disease context-specific target gene or protein modification profiles. Within tumor microenvironments, KL-la preferentially upregulates antioxidant and iron homeostasis-associated targets such as through H3K18la-mediated indirect GPX4 enhancement to diminish LCD susceptibility, contrasting sharply with non-tumor pathologies such as non-alcoholic steatohepatitis wherein KL-la preferentially modifies pro-death or stress-associated targets such as through H3K18la-mediated indirect GPX4 suppression to amplify LCD and intensify pathological progression.

Given profound KL-la-mediated LCD regulatory context-dependence, therapeutic strategies targeting the lactate-lactylation axis necessitate precision and innovation. Current approaches bifurcate into two principal categories wherein the first aims at lactate generation suppression through chemical drugs, traditional Chinese medicines, or gene therapies targeting glycolytic nodes including PKM2, PDK4, and LDHA to diminish lactate accumulation and intervene in downstream KL-la and LCD processes. This category exhibits constraints wherein chemical pharmaceuticals and traditional Chinese medicines predominantly target PKM2 with insufficient exploration of alternative critical targets including LDHA and PFKFB3 alongside narrow disease spectra, while gene therapies primarily target LDHA within non-tumor pathologies with tumor and alternative target efficacy validation remaining outstanding. The second category directly targets lactylation modifying enzymes through lactyl transferase such as KAT8 or delactylase such as HDAC1 and SIRT1 activity modulation for precise lactylation level regulation, though current investigations exhibit limited target coverage with sparse exploration of broader enzymatic spectra including HDAC2/3, SIRT2/3, and KAT2, alongside notably insufficient tumor model therapeutic validation. To surmount these constraints and achieve safe, effective interventions, future therapeutic development must pursue precision and intelligent paradigms. Network pharmacology, molecular docking, and molecular dynamics simulation computational platforms should systematically screen and design novel agents targeting axis-critical nodes. Crucially, context-responsive precision intervention strategies must be developed based on KL-la regulation-dependent disease backgrounds, cellular identities, and specific target proteins, encompassing tissue or cell type-targeted delivery systems alongside prodrugs or context-responsive inhibitors activating under specific pathological microenvironments such as acidic pH or lactate-enriched conditions to confine therapeutic effects within target pathological cells, maximally reducing off-target toxicities while broadening therapeutic windows to ultimately achieve safe, effective axis modulation.

Current mechanistic understanding of lactylation modification-mediated LCD regulation influencing disease progression, despite progress, remains constrained by methodological limitations. Detection and validation face specificity challenges wherein prevailing L-lactylation modification detection methods including antibody-based approaches exhibit cross-reactivity with structurally analogous lysine acylation modifications such as acetylation and lactylation isomers such as KD-la, while mass spectrometry encounters difficulties distinguishing closely related mass-to-charge ratio modification isomers, compromising result comparability. Establishing definitive cause-function relationships for lactylation modifications presents challenges as current investigations predominantly rely on correlational analyses such as lactate level and modification level covariation to infer functionality, inadequately demonstrating specific modifications causally drive phenotypic alterations. Moreover, traditional irreversible site mutation approaches such as lysine-to-arginine substitutions for functional site identification, while erasing target lactylation, may disrupt additional site functionalities including acetylation modifications, confounding precise lactylation site functional determination (7). Consequently, it is imperative to thoroughly consider the impact of methodological limitations when interpreting the results of existing studies. Specifically, the clinical translation of therapeutic strategies that target lactic acid metabolism to regulate LCD should be approached with caution. Looking ahead, the advancement of reversible and site-specific manipulation technologies, such as genetic code expansion technology (103) bioorthogonal chemical reporting technology (104) holds promise for overcoming the current methodological bottlenecks.

In conclusion, lactate as a pivotal metabolic signaling molecule orchestrates sophisticated lytic cell death regulatory networks at epigenetic and post-translational tiers through histone and non-histone lactylation modification mediation. This review systematically delineates core KL-la-LCD-disease regulatory axis characteristics wherein KL-la achieves bidirectional regulation of LCD critical molecules with effects exhibiting profound context-dependence as functionality is jointly determined by disease-specific metabolic profiles, inflammatory states, and cellular identities, while axis targeting furnishes novel therapeutic avenues. Nonetheless, several critical issues remain to be addressed in the current research. Firstly, the comprehensive molecular network of KL-la in the regulation of LCD has yet to be fully elucidated. Secondly, it remains unclear whether there is competition or synergy between KL-la and other post-translational modifications, such as acetylation, succinylation, and ubiquitination, in the regulation of LCD. Thirdly, the potential synergy between histone and non-histone lactylation modifications requires further investigation. Additionally, it is necessary to explore whether the bidirectional regulation of KL-la on LCD is dynamically modulated by the “writing enzyme-erasing enzyme” system, beyond the context of disease background. Current research on the “lactylation modification-LCD-disease” axis predominantly concentrates on KL-la, with limited attention given to other lactate-related modifications such as KD-la and Kce. Future investigations should aim to elucidate the roles of KD-la and Kce in the pathogenesis and progression of LCD, as well as their influence on the disease process. It is also imperative to determine whether KL-la, KD-la, and Kce collectively contribute to the modification processes within LCD. Advancing the understanding of these scientific questions will enhance the comprehension of the intrinsic relationship between lactic acid metabolism and cell fate determination, thereby providing a crucial theoretical foundation for the development of precision therapeutic strategies.
