# Supplementary material for: Role of lactylation modification in regulating lytic cell death
Source: Front Oncol. 2026 Jan 27;16:1718636. doi: 10.3389/fonc.2026.1718636 (PMC12887707; doi:10.3389/fonc.2026.1718636)
Supplement: Supplementary file 1 [file Table1.doc]

**Supplementary Material(s)**

**Supplementary Table 1 Regulatory Enzymes Involved in Protein Lactylation Modifications**

| Functional Classification   |  | | --- | |  | | Target Protein | Research Group | Reference |
| --- | --- | --- | --- |
| Lactyl-CoA synthetases | ACSS2 | Zhu et al | (40) |
|  | GTPSCS | Liu et al | (41) |
| Lactyltransferases (writers) | P300 | Zhang et al, Wang et al | (42,43) |
|  | CBP | Chen et al | (44) |
|  | GCN5 | Wang et al | (45) |
|  | MOF | Xie et al, Yuan et al | (46,47) |
|  | TIP60 | Chen et al | (48) |
|  | HBO1 | Niu et al | (49) |
|  | AARS1/2 | Ju et al, Zong et al, Li et al, Mao et al, | (50-53) |
|  | HDAC6 | Sun et al | (54) |
| Delactylases (erasers) | HDAC1-3 | Gao et al, Chen et al, He et al | (38,48,55) |
|  | SIRT1-3 | Fan et al, Wang et al | (56,57) |
| Lactylation recognition proteins (readers) | Brg1 | Hu et al | (58) |
|  | DPF2 | Zhai et al | (59) |
|  | TRIM33 | Nuñez et al | (60) |

**Abbreviation:** H3K18: histone H3 lysine 18, H4K12: histone H4 lysine 12, H3K14: histone H3 lysine 14, HMGB1: high-mobility group box 1, NOD2: nucleotide-binding oligomerization domain-containing 2, YTHDF3: YTH N6-methyladenosine RNA-binding protein 3, ALKBH5: AlkB homolog 5 RNA demethylase, NLRP3: NOD-like receptor family pyrin domain-containing 3, NEK7: NIMA-related kinase 7, ATF3: activating transcription factor 3, ATF4: activating transcription factor 4, CHAC1: ChaC glutathione-specific gamma-glutamylcyclotransferase 1, ACSL4: acyl-CoA synthetase long-chain family member 4, METTL3: methyltransferase-like 3, AIM2: absent in melanoma 2, NSF1: N-ethylmaleimide-sensitive factor 1, ZFP64: zinc finger protein 64, HIF1α: hypoxia-inducible factor 1 alpha, GCLC: glutamate-cysteine ligase catalytic subunit, TFRC: transferrin receptor. CIRI: cerebral ischemia–reperfusion injury, BRE: bilirubin encephalopathy, SEV-CI: sevoflurane-induced cognitive impairment, GSD-IBD: glycogen storage disease–associated inflammatory bowel disease, UC: ulcerative colitis, AD: Alzheimer’s disease, NASH: nonalcoholic steatohepatitis, IVDD: intervertebral disc degeneration, SILI: sepsis-induced lung injury, LC: lung cancer, HCC: hepatocellular carcinoma, TNBC: triple-negative breast cancer, PCa: prostate cancer, CRC: colorectal cancer, SI-ARDS: sepsis-induced acute respiratory distress syndrome, SAKI: sepsis-associated acute kidney injury.
